# Biosynthesis of a Novel Diketopiperazine Aspkyncin Incorporating a Kynurenine Unit from *Aspergillus aculeatus*

**DOI:** 10.3390/jof11030171

**Published:** 2025-02-20

**Authors:** Dekun Kong, Xin Wang, Li Liu

**Affiliations:** 1 College of Pharmaceutical Sciences, Southwest University, Chongqing 400715, China; wx19980822@email.swu.edu.cn; 2 Laboratory of Biochemistry and Molecular Biology, Lab Teaching and Management Center, Chongqing Medical University, Chongqing 400016, China

**Keywords:** diketopiperazine, fungi, l-kynurenine, *N*-methyltransferase, non-ribosomal peptide synthetase (NRPS)

## Abstract

The simplest cyclo-peptides, also known as diketopiperazines (DKPs), are widespread in nature. The growing interest in these simplest cyclo-peptides is driven by their significant potential for therapeutic applications. In this study, we identified a biosynthetic gene cluster from *Aspergillus aculeatus* CRI323-04 through genome mining and heterologous expression in *Aspergillus nidulans*. The two core genes, *aacA* and *aacB*, within the gene cluster were characterized for their role in the biossoynthesis of aspkyncin, a novel DKP compound that incorporates a l-kynurenine (l-Kyn) unit. Furthermore, we successfully reconstituted the activities of the minimal bimodular non-ribosomal peptide synthetase (NRPS) AacA and the methyltransferase AacB both in vivo and in vitro. Our findings demonstrate that AacA catalyzes the condensation and cyclization of two non-proteinogenic amino acids, l-Kyn and *N*-methyl-l-alanine, to produce aspkyncin without the involvement of any release domain. Notably, the *N*-methyl-l-alanine is generated by a specialized l-alanine *N*-methyltransferase AacB prior to NRP assembly. This study reveals an unconventional pathway for the biosynthesis of fungal DKPs.

## 1. Introduction

**Figure 1:**
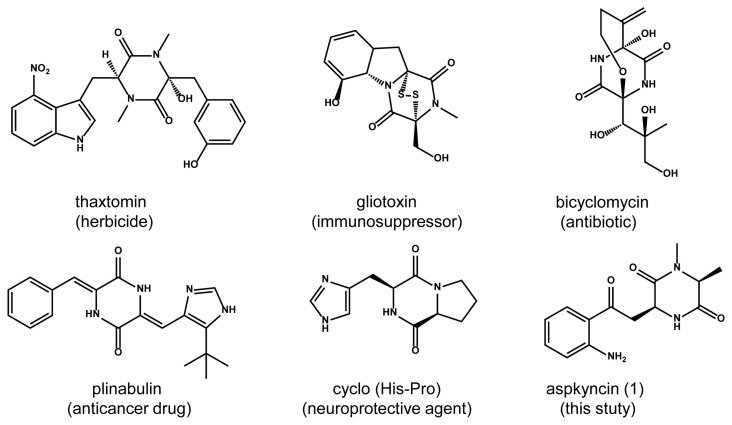
The structures and activities of representative 2,5-diketopiperazines.

2,5-diketopiperazines (2,5-DKPs) are cyclo-dipeptides formed by the condensation of two α-amino acids (Figure 1) [1,2,3]. These compounds, which are present in the metabolites of bacteria, fungi, plants, and mammals, demonstrate a wide range of biological activities, such as anticancer, antioxidant, and neuroprotective effects [4]. Compared with traditional linear peptides, DKPs exhibit several significant chemical advantages, including the ability to mimic pharmacological groups of polypeptides, enhanced enzymatic stability, and increased structural rigidity [5]. These characteristics make DKPs an ideal scaffold for drug discovery. The growing interest in these simple natural cyclo-peptides is evident due to their significant potential in therapeutic applications. Based on biosynthetic mechanisms, DKPs can be categorized into three main groups: in mammals, the DKP cyclo (l-His-l-Pro) is derived from the nonenzymatic cyclization of thyrotropin-releasing hormone (TRH, pGlu-His-Pro) after cleavage by pyroglutamate aminopeptidase; *Streptomyces noursei* utilizes cyclodipeptide synthases to produce albonoursin; and fungi mainly utilize non-ribosomal peptide synthetase (NRPS) for DKP biosynthesis [6,7,8].

So far, no DKP containing the l-Kynurenine (l-Kyn) unit has been reported in fungi. Kyn is a critical intermediate in tryptophan metabolism, with over 95% of dietary tryptophan being metabolized through the Kyn pathway to generate nicotinamide and NAD^+^. Kyn and its derivatives interact with various tissue-specific receptors, influencing inflammation, immune responses, and excitatory neurotransmission. It has also emerged as a key factor in diabetes and cancer [9,10]. The bioavailability of Kyn can be modulated by the gut microbiome, where resident microorganisms can directly absorb tryptophan, thereby limiting its availability to the host. The resulting metabolites can exert local effects on both the microbiome and host cells. As the roles of Kyn metabolites continue to be elucidated in various physiological and disease contexts, the discovery of novel Kyn analogs is expected to offer clinical benefits in multiple human disorders, serving as potential targets for drug discovery and development [11].

During the investigation of secondary metabolite synthesis in *Aspergillus aculeatus* CRI323-04, we identified a biosynthetic gene cluster, designated as *aac*, encoding an atypical bimodular NRPS. This NRPS is distinguished by its minimal domain architecture, comprising two adenylation (A) domains, one condensation (C) domain, and two thiolation (T) domains, arranged in the order of A-T-C-A-T. In this study, we report the structural elucidation and biosynthesis of a novel DKP compound, named aspkyncin, which features a l-Kyn unit (Figure 1). The biosynthetic pathway of aspkyncin was elucidated through heterologous expression in *Aspergillus nidulans* and in vitro biochemical characterization. The minimal bimodular NRPS, which contains no release domain, catalyzed the synthesis of aspkyncin. Additionally, we discovered a novel proteinogenic amino acid *N*-methyltransferase that uses l-alanine as a substrate.

## 2. Materials and Methods

### 2.1. Strains and Culture Conditions

*Aspergillus aculeatus* CRI323-04 strain (obtained from Professor Prasat Kittakoop’s laboratory at Chulabhorn Graduate Institute) was grown at 25 °C for 7 days in PDB medium (6 g/L potato infusion powder, 20 g/L glucose) for gDNA extraction [12]. *Saccharomyces cerevisiae* BJ5464 was used for homologous recombination to construct plasmids. *S. cerevisiae* was grown at 30 °C in YPD medium (20 g/L glucose, 20 g/L tryptone, 10 g/L yeast extract). *Aspergillus nidulans* LO8030 (genotype: *pyrG89*; *pyroA4*; *nkuA::argB*; *riboB2*) was used as the host for heterologous expression and protein expression [13]. *A. nidulans* was cultured at 37 °C for 3 days on solid GMM medium (10 g/L D-glucose, 6 g/L NaNO_3_, 0.52 g/L KCl, 0.52 g/L MgSO_4_·7H_2_O, 1.52 g/L KH_2_PO4, 15 g/L agar, and 1 mL/L Hutner’s trace element solution) for sporulation or at 25 °C for 2.5 days in liquid modified GMM medium (20 g/L starch, 10 g/L casein hydrolysate, 6 g/L NaNO_3_, 0.52 g/L KCl, 0.52 g/L MgSO_4_·7H_2_O, 1.52 g/L KH_2_PO_4_, and 1 mL/L Hutner’s trace element solution) for heterologous protein expression. *Escherichia coli* BL21 was used for the protein expression of AacB. *E. coli* strains were cultured at 37 °C for cloning or 25 °C for protein expression and grown in LB medium supplemented with the respective antibiotics.

### 2.2. General DNA Manipulation Techniques

The genomic DNA of *A. aculeatus* CRI323-04 was extracted using the CTAB method [14]. DNA restriction enzymes were used as recommended by the manufacturer (New England Biolabs, Ipswich, MA, USA). PCR reactions were performed using Phanta Max Super-Fidelity DNA Polymerase (Vazyme, Nanjing, China). The gene-specific primers are listed in Table S2 in the Supplementary Materials. The plasmids (Table S3 and Figure S1 in the Supplementary Materials) that were used for heterologous expression in *A. nidulans* were constructed by yeast homologous recombination using the Frozen-EZ Yeast Transformation II^TM^ Kit (Zymo Research, Irvine, CA, USA). The yeast plasmid extraction was performed with Zymoprep^TM^ Yeast Plasmid Miniprep I Kit (Zymo Inc., Irvine, CA, USA). Sangon Biotech Co., Ltd. (Shanghai, China) carried out the synthesis of primers and DNA sequencing.

### 2.3. Plasmids Construction

The vectors employed are pANP, pANU, and pANR, with auxotrophic markers for pyridoxine (pyroA), uracil (pyrG), and riboflavin (riboB), respectively. Target genes were inserted into pAN vectors, and the resulting recombinant plasmids were then transferred into *A. nidulans* to construct mutants. The target genes were amplified from the gDNA of *A. aculeatus* CRI323-04, with their native terminators added to the 3′ ends. The prepared fragments and vectors were co-transferred into *S. cerevisiae* to yield the plasmids for gene expression in *A. nidulans*.

To construct the protein expression plasmid of AacB for *E. coli*, the intron-free *aacB* gene was amplified from the cDNA of the *A. nidulans* strain heterologously expressing the *aac* gene cluster, using the primers listed in Table S2, cloned into expression pGEX-4T-1 vector digested with *Eco*RI and *Not*I.

To construct a protein expression plasmid of AacA for *A. nidulans*, an 8 × His-tag sequence was fused at the 3′ end using PCR. The gene with 8 × His-tag and its terminator (611 bp) were amplified from gDNA of *A.s aculeatus* CRI323-04. These fragments were then cloned into the pANR vector digested with *Not*I.

### 2.4. Protoplast Preparation and Transformation of A. nidulans

The 10^9^ fresh spores (2 × 10^6^ spores/mL, about half plate spores) of *A. nidulans* were inoculated in 50 mL liquid GMM medium containing 10 mM uridine, 5 mM uracil, 0.5 μg/mL pyridoxine, and 0.125 μg/mL riboflavin and cultured at 37 °C and 220 rpm for 9 h. Following spore germination, the cultures were subjected to centrifugation at 4 °C and 3301× *g* for 8 min to collect the mycelia, which were subsequently washed twice with 15 mL of OM buffer (1.2 mol/L MgSO_4_·7H_2_O, 10 mM sodium phosphate, pH 5.8) at 4 °C and 3750 rpm for 8 min each time. A total of 10 mL OM buffer containing 30 mg of Lysing enzyme from *Trichoderma harzianum* (Sigma-Aldrich, St. Louis, MO, USA) and 20 mg Yatalase (Takara, Kusatsu, Japan) was used to digest the germlings at 28 °C, 80 rpm for 10 h. The digested mixture was then added to 10 mL trapping buffer (0.6 M sorbitol, 0.1 M Tris-HCl, pH 7.0) on ice and centrifuged at 4 °C, 3750 rpm for 20 min to collect the protoplasts from the middle layer. The protoplasts were transferred and fully dispersed into a twofold volume of STC buffer (1.2 M sorbitol, 10 mM CaCl_2_, 10 mM Tris-HCl, pH 7.5) and centrifuged at 4 °C, 3750 rpm for 10 min. The supernatant was removed and added with STC buffer at a concentration of 10^8^–10^9^ cells/mL (usually 1 mL) to resuspend the protoplasts for transformation.

For transformation, 2 μg of each recombinant plasmids were added to the 100 μL protoplasts of *A. nidulans* and placed on ice for 30 min. A total of 600 μL 60% PEG buffer (60% PEG 4000, 50 mM CaCl_2_, 50 mM Tris, pH 7.5) was added to the mixture and then incubated at room temperature for 1 h. Protoplasts were directly plated on solid sorbitol-stabilized agar plates (GMM medium with 1.2 mM sorbitol and appropriate supplements) at 37 °C for 2 days. The transformants were inoculated on fresh dropout solid medium at 37 °C for 3 days.

### 2.5. Chemical Analysis and Compound Isolation and Characterization

LC-MS analysis was performed on Waters ACQUITY H-Class UPLC-MS system coupled to a PDA detector and an SQD2 mass spectrometer (MS) detector with an ESI source. Chromatographic separation was executed at 35 °C utilizing a C18 column (ACQUITY UPLC^®^ BEH, 1.7 μm, 2.1 mm × 100 mm, Waters, Milford, MA, USA). LC-MS metabolite profiles were generated with the method outlined below: a linear gradient of 5–99% MeCN-H_2_O (both containing 0.02% *v*/*v* formic acid) in 10 min followed by 99% MeCN-H_2_O for 3 min and then 5% MeCN-H_2_O for 3 min at a flow rate of 0.4 mL/min. The MS data were collected in the *m*/*z* range of 50–1500 in positive mode. MPLC was performed on the BUCHI Reveleris^®^ X2 Flash Chromatography System (Buchi, Flawil, Switzerland), with UV detectors and a BUCHI Reveleris^®^ C18 column (40 μm, 80 g). Semi-preparative HPLC was performed on a Shimadzu Prominence HPLC system (Shimadzu, Kyoto, Japan) using a YMC-Pack ODS-A column (5 μm, 10 × 250 mm). NMR spectra were recorded on a Bruker AVANCE III NMR 400 MHz (Bruker, Bielefeld, Germany) with a 5 mm broadband probe and TMS as an internal standard. HRMS data were generated using a quadrupole time-of-flight (Q-TOF) mass spectrometer (Bruker IMPACT II, Bruker, Bremen, Germany).

For the isolation of compound aspkyncin, the plasmid pIM2515 was introduced into *A. nidulans* to construct *A. nidulans*-*aacAB* strain. The *A. nidulans*-*aacAB* strain was cultivated on 5 L modified GMM agar medium (50 petri dishes, diameter 80 cm) at 25 °C for 3.5 days. The cultures were then extracted with a mixture of ethyl acetate and acetone (*v*/*v* = 3/1) three times. The resulting upper organic solvent was evaporated to dryness under vacuum to obtain the residues. The residues then underwent a purification regimen via MPLC with 30% MeOH-H_2_O for 15 min and then a linear gradient of 30% to 55% MeOH-H_2_O for 40 min at a flow rate of 25 mL/min. The fraction containing compound aspkyncin was purified by semi-preparative HPLC using the isocratic program of 28% MeOH-H_2_O to afford compound aspkyncin (3.0 mg, t_R_ = 37.5 min).

### 2.6. Expression and Purification of AacA and AacB

To purify soluble AacA with 8 × His-tag, the plasmid pIM2513 was introduced into *A. nidulans* to construct *A. nidulans*-*aacA* (with 8 × His-tag) strain. Spores of *A. nidulans*-*aacA* (with 8 × His-tag) were inoculated in 500 mL liquid modified GMM medium and cultured at 25 °C and 220 rpm for 2.5 days. The culture medium was filtered through gauze to collect the mycelium for protein purification. The mycelium was quick-frozen with liquid nitrogen and ground into a fine powder using a ball mill then resuspend in 100 mL buffer A (50 mM Tris-HCl, 500 mM NaCl, 10% glycerol, pH 7.5). The suspension was centrifuged at 24,470× *g* at 4 °C for 30 min. The supernatant was incubated with nickel nitrilotriacetic acid (Ni-NTA) agarose resin. After removing miscellaneous proteins using buffer A containing 50 mM imidazole, the His-tagged protein was eluted using buffer A containing 250 mM imidazole. The elute was passed through a PD-10 desalting column (GE Healthcare, Chicago, IL, USA), and the purified protein was eluted with buffer C (50 mM Tris-HCl, 50 mM NaCl, 5% glycerol, pH 7.5). The protein was concentrated to a volume of 200 μL using an ultrafiltration centrifugal tube (Millipore Amicon^®^ Ultra-15 mL, Burlington, MA, USA). Finally, the protein was flash-frozen in liquid nitrogen and stored at −80 °C.

To purify soluble AacB with an *N*-GST-tag, the plasmid pIM2562 was introduced into *E. coli* BL21 strain and grown in liquid LB medium at 37 °C with shaking at 220 rpm until reaching an OD_600_ of 0.4–0.6. The cultures were kept on ice for 10 min. Subsequently, the cultures were induced with 0.2 mM isopropylthio-β-D-galactoside (IPTG) at 25 °C with shaking at 220 rpm for 8 h. The cells were pelleted by centrifugation at 4 °C, 6118× *g* for 5 min, resuspended in PBS buffer (140 mM NaCl, 2.7 mM KCl, 10 mM Na_2_HPO_4_, 1.8 mM KH_2_PO_4_, pH 7.3), and lysed by sonication on ice. Then, the supernatant was obtained by centrifugation at 4 °C, 24,470× *g* for 40 min and incubated with Glutathione Sepharose 4B resin (GE Healthcare, Chicago, IL, USA). The resin was washed with PBS buffer to remove non-specific proteins, the GST-tagged protein was eluted by GST elution buffer (50 mM Tris-HCl, 10 mM reduced glutathione, pH 8.0). The pooled fraction was concentrated and exchanged into buffer C (50 mM Tris-HCl, 50 mM NaCl, 5% glycerol, pH 7.5). The purified enzyme was analyzed by SDS-PAGE, and the concentration was measured with a BCA protein quantification kit (Beijing Dingguo Changsheng Biotechnology Co., Ltd., Beijing, China).

### 2.7. In Vitro Enzymatic Assays of AacA and AacB

For the in vitro assay of AacA, the reaction was conducted in 50 μL buffer C (pH 7.5), containing 10 μM AacA, 100 μM l-alanine/*N*-methyl-l-alanine, 100 μM l-Kyn, 1 mM ATP and 1 mM MgCl_2_. For the in vitro assay of AacA and AacB, the reaction was conducted in 50 μL buffer C (pH 7.5), containing 10 μM AacA, 10 μM AacB, 1 mM SAM, 100 μM l-alanine, 100 μM l-Kyn, 1 mM ATP, and 1 mM MgCl_2_. The in vitro assays were incubated at 25 °C for 6 h then stopped by the addition of twice the volume of ethyl acetate. The extracted ethyl acetate layer was then evaporated to dryness and re-dissolved in 100 μL methanol.

For the in vitro assay of AacB, the reaction was conducted in 100 μL buffer C (pH 7.5), containing 10 μM AacB, 1 mM SAM, and 200 μM l-alanine at 25 °C for 5 h. Then, 20 μL 1 M NaHCO_3_ and 100 μL 1% (*w*/*v*) 1-fluoro-2,4-dinitrophenyl-5-l-leucinamide (l-FDLA) in acetone were added in the reaction mixtures and incubated at 45 °C for 2 h. The reaction mixtures were cooled to room temperature and the reaction was quenched by adding 10 μL 2 N HCl. The reaction mixtures were dried, dissolved in 300 μL 50% aqueous MeCN to yield FDLA derivatives, and analyzed by LC-MS. The standard amino acids l-alanine and *N*-methyl-l-alanine were also treated with the same method.

### 2.8. RT-PCR of acc Cluster Genes

For RT-PCR analysis, total RNA was extracted from *A. aculeatus* CRI323-04 cells using a RNeasy kit (Qiagen, Hilden, Germany) according to the manufacturer’s instructions. cDNA for each sample was synthesized using 1.0 μg of total RNA and Superscript III reverse transcriptase with oligo (dT) priming following the manufacturer’s protocol (Invitrogen, Carlsbad, CA, USA). Amplification of portions of the *gapdh* gene was performed as controls and internal standards.

### 2.9. Bioinformatics Analysis

Gene cluster annotation was performed using the 2ndFind program to predict open reading frames and introns, and gene functions were assigned based on BlastP search results. Multiple sequence alignments of the A domain and its homologous proteins were conducted by DNAMAN 8.0 software. The domains of the core genes were analyzed by the interpro website. Adenylation (A) domain codes for NRPSs substrate recognition were analyzed with PKS/NRPS Analysis website. 2ndFind: http://biosyn.nih.go.jp/2ndFind/ (accessed 8 September 2023); NCBI BLAST: https://blast.ncbi.nlm.nih.gov/Blast.cgi (accessed 8 September 2023); PKS/NRPS Analysis: https://nrps.igs.umaryland.edu/index.html (accessed 16 November 2023).

## 3. Results

### 3.1. Bioinformatic Analysis of the Biosynthetic Gene Cluster

**Figure 2:**
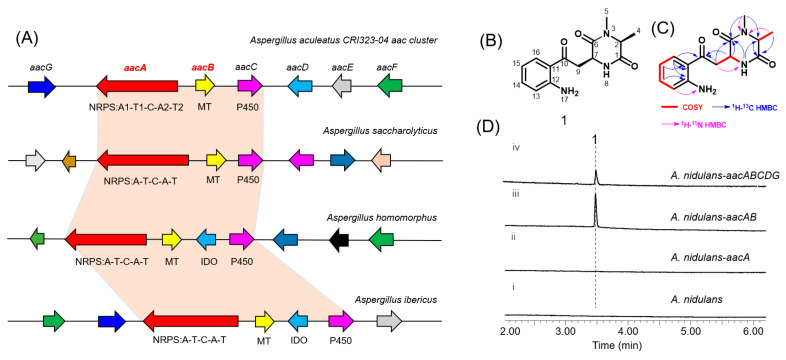
(**A**) Schematic representation of the *aac* cluster and homologous gene clusters in *Aspergillus*. AacA: NRPS; AacB: methyltransferase (MT); AacC: cytochrome P450, AacD: amino-acid oxidase; AacE: hypothetical protein; AacF: transcription factor; AacG: carboxylesterase; IDO: indoleamine-2,3-dioxygenase. (**B**) Structure of 1 (aspkyncin). (**C**) ^1^H−^1^H COSY and HMBC correlations in aspkyncin. (**D**) In vivo reconstitution of *aac*. Shown is HPLC analysis (λ = 366 nm) of metabolites extracted from 3-day cultures of (i) untransformed *A. nidulans* LO8030, (ii) *A. nidulans* LO8030 expressing AacA, (iii) *A. nidulans* LO8030 expressing AacAB, and (iv) *A. nidulans* LO8030 expressing AacABCDG.

The *aac* cluster comprises seven contiguous genes (*aacA-G*, NCBI accession number PV107498) which code for an NRPS (AacA), a methyltransferase (AacB), a cytochrome P450 oxygenase (AacC), an amino-acid oxidase (AacD), a hypothetical protein (AacE), a transcription factor (AacF), and a carboxylesterase (AacG) (Figure 2A, Table S1). Homology-based annotation of AacA reveals the presence of two adenylation (A) domains, one condensation (C) domain, and two thiolation (T) domains, arranged as A1-T1-C-A2-T2. Notably, AacA represents an atypical NRPS, distinguished by the absence of terminal cyclization and release domains such as thioesterase (TE), reductase (R), and additional C_T_ domains [15]. By using the core biosynthetic enzymes AacA and AacB as queries, we identified a series of homologous gene clusters widely distributed across *Aspergillus* species (Figure 2A). The conserved orthologs include NRPS, methyltransferase, and cytochrome P450. Importantly, two of these homologous clusters contain an indoleamine-2,3-dioxygenase (IDO). In fungi, IDO catalyzes the conversion of l-tryptophan to *N*-formyl-l-kynurenine, which is rapidly hydrolyzed to l-Kyn [16]. These findings strongly suggest that the *aac* cluster may be involved in the biosynthesis of novel NRP natural products incorporating a l-Kyn unit.

First, we aim to identify the compound(s) produced by the gene cluster. Reverse transcriptase-polymerase chain reaction (RT-PCR) analysis of total RNA from *A. aculeatus* CRI323-04 failed to detect transcription of the *aac* cluster genes, indicating that this gene cluster is silent under standard laboratory culturing conditions. To activate the biosynthetic pathway, we attempted to overexpress a putative zinc finger domain-containing transcription factor encoded by *aacF*, a strategy that has recently been successfully employed in several fungal species [17]. However, this approach did not result in any observable changes in the metabolic profile (Figure S2).

### 3.2. Heterologous Expression of the aac Cluster and Characterization of the Products

To explore the natural product encoded by the *aac* cluster, all five putative biosynthetic genes except *aacF* were introduced into an engineered *A. nidulans* expression host on three replicative vectors containing the *A. nidulans* FGSC A4-derived AMA1 replicon (Table S3 and Figure S1). Compared to the negative control (Figure 2D, trace i), a new metabolite, compound 1, with a *m*/*z* of 275, was identified from the extract of *A. nidulans* expressing *aacABCDG* (Figure 2D, trace iv). To identify the genes involved in the biosynthesis of compound 1, different combinations of genes from the *aac* cluster were co-expressed in *A. nidulans*. Interestingly, only *aacA* and *aacB* were required to catalyze the synthesis of compound 1 (Figure 2D, trace iii).

Subsequent large-scale fermentation, purification, and structural determination by HR-MS and NMR analyses (Figures S9–S29, Table S4) confirmed that compound 1 is 3-(2-(2-aminophenyl)-2-oxoethyl)-1,6-dimethylpiperazine-2,5-dione, and it was named as aspkyncin (Figure 2B,C). Characterization of aspkyncin validated our hypothesis that the *aac* cluster encodes enzymes responsible for the biosynthesis of a natural product belonging to the DKP alkaloids. Retrosynthetic analysis of the compound aspkyncin suggests that the DKP scaffold originates from the nonproteinogenic amino acid l-Kyn, which forms an amide bond with either *N*-methyl-l-alanine or l-alanine. However, l-Kyn is rarely encountered as a building block or biosynthetic precursor in bioactive natural products. Over the past few decades, only a limited number of examples have been reported. Recent studies have revealed that l-Kyn can be incorporated via NRPS biosynthetic pathways to generate 1-benzazepine-containing compounds [18,19]. Additionally, the antibiotic daptomycin has been shown to incorporate l-Kyn as a building block (Figure S3) [20].

Adenylation domains of NRPS select the monomers to be incorporated into the natural product. The amino acid residues occupying ten key positions are collectively referred to as the Stachelhaus code [21,22]. For fungal A domains, substrate prediction is significantly less accurate compared to bacterial counterparts. The NRP codes of AacA in A1 (D_217_A_218_L_221_F_258_V_279_G_281_G_302_I_310_G_311_K_501_) and A2 (D_217_I_218_A_221_C_258_V_279_A_281_L_302_I_310_L_311_R_503_) exhibit limited conservation relative to the previously reported A domain code for l-Kyn [19,23]. Notably, arginine replaces the relatively conserved lysine at the 10th position in the two reported A domains involved in l-Kyn recognition. Additionally, the A1 code shows certain similarities with the previously reported alanine-specific A domain [24]. Based on these observations, it is hypothesized that the A2 domain of AacA is responsible for recognizing l-Kyn (Figure S4).

### 3.3. Characterization of AacB as an l-Alanine N-methyltransferase

When *aacA* was heterologously expressed alone in *A. nidulans*, no production of aspkyncin was detected, indicating that l-alanine methylation is essential for the synthesis of the product. The *N*-methylation of l-Alanine can occur through three mechanisms: (1) direct methylation on the amino acid; (2) methylation on the NRP assembly line; and (3) methylation on the cyclized piperazine diketone [8]. The second mechanism is the most common, as exemplified by TxtA, and TxtB [2]. However, the heterologous expression results of aspkyncin suggest the possibility of the first mechanisms, because there is no methyltransferase domain in AacA, and no demethylated intermediates were detected when *aacA* was expressed alone (Figure 2D, trace ii). To test this hypothesis, we initially performed in vivo feeding experiments. Feeding *N*-methyl-l-alanine to an *A. nidulans* strain heterologously expressing *aacA* led to the production of aspkyncin (Figure S5, trace ii), whereas supplementation with l-alanine did not yield any detectable product (Figure S5, trace iii). These results suggest that l-alanine methylation serves as the initial step in the biosynthesis of aspkyncin, and AacB acts as a specific l-alanine methyltransferase.

**Figure 3:**
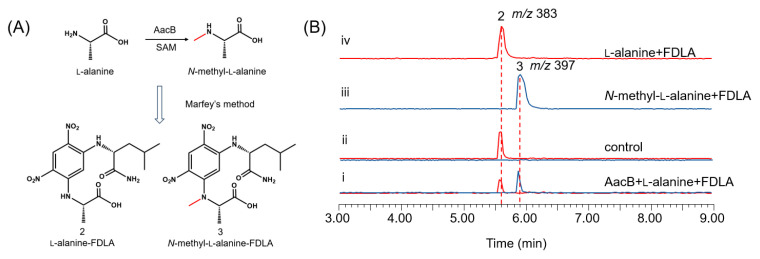
Characterization of AacB: (**A**) amino acid derivatization diagram; (**B**) in vitro reconstitution of AacB. Shown are LC-MS analyses of compounds from the extraction of reaction mixtures after Marfey’s method of (i) AacB + l-alanine, (ii) no enzyme control, (iii) *N*-methyl-l-alanine, and (iv) l-alanine.

To further validate the function of AacB, the intron-free AacB gene was cloned from cDNA extracted from *A. nidulans* expressing the *aac* cluster and overexpressed in *E. coli* BL21 as a GST-tagged fusion protein (Figure S6). The reaction was carried out in 50 mM Tris-HCl buffer (pH 7.5) with the addition of AacB, l-alanine, and the cofactor S-adenosylmethionine (SAM), and then the reaction mixture was derivatized using the Marfey’s method. The methylated product, *N*-methyl-l-alanine-FDLA, was detected (Figure 3B, trace i). These experimental results provide strong evidence that AacB functions as an *N*-methyltransferase for l-alanine.

### 3.4. In Vitro Reconstitution of the AacA Activities

So far, only a few NRPSs without release domains have been reported, such as AnaPS (C*-A1-T1-C-A2-T2-E), which synthesizes acetylaszonalenin [25]. The in vivo heterologous expression results indicate that AacA can catalyze the condensation of l-Kyn and *N*-methyl-l-alanine to form the DKP structure. However, it remains uncertain as to whether AacA, lacking the release domain, can independently synthesize aspkyncin, because there are several possibilities for the release of this product, including type II TEs-catalyzed hydrolysis and spontaneous hydrolysis [15].

**Figure 4:**
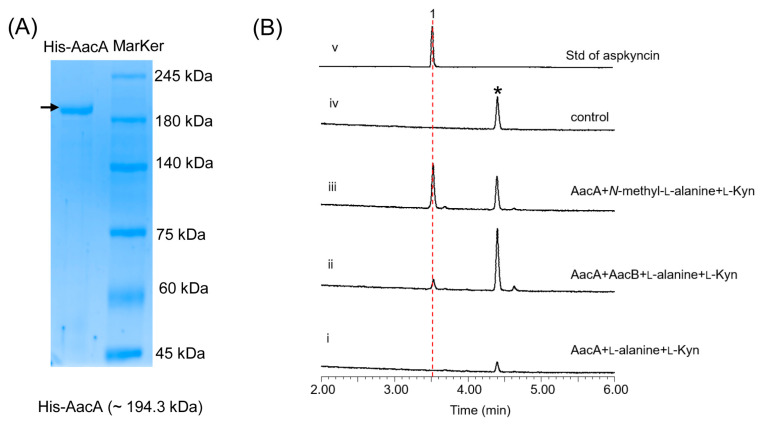
Characterization of AacA: (**A**) SDS-PAGE analyses of AacA (~194.3 kDa) with 8 × His-tag; (**B**) in vitro reconstitution of AacA. Shown are HPLC analyses (λ = 366 nm) of compounds from the extraction of reaction mixtures of (i) AacA + l-alanine + l-Kyn; (ii) AacA + AacB + l-alanine + l-Kyn; (iii) AacA + *N*-methyl-l-alanine + l-Kyn; (iv) no enzyme control (only *N*-methyl-l-alanine + l-Kyn in reaction buffer); and (v) standard of aspkyncin.

To identify the AacA involved in the cyclization of linear peptides, we first sought to reconstitute the activity of NRPS in vitro. To achieve this, we constructed *A. nidulans* replicative vectors carrying the gene encoding AacA (5.2 kb), with an 8 × His-tag fused to its C-terminus, under the control of the *gpdA* promoter. The full-length AacA (194 kDa) was then purified to near homogeneity using Ni-NTA affinity chromatography (Figure 4A). We subsequently performed a complete reconstitution of AacA activity by adding amino acid building blocks (100 μM each) to the purified enzyme (10 μM). The reaction mixtures were incubated at 25 °C for 12 h, extracted with ethyl acetate, and analyzed by LC-MS. Results show that, when *N*-methyl-l-alanine and l-Kyn were used as the substrates, aspkyncin was synthesized as the sole peptidyl product (Figure 4B, trace iii). When l-alanine was used as the substrate, no product was generated, further indicating that methylation of l-alanine occurs prior to NRP assembly (Figure 4B, trace i).

**Figure 5:**
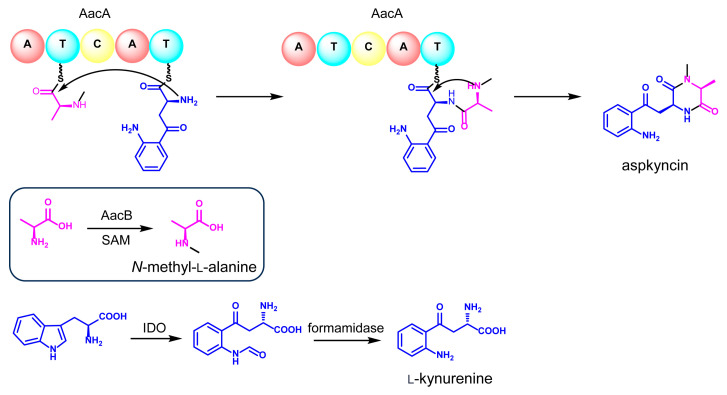
The proposed biosynthetic pathway of aspkyncin.

Reconstitution of the synthesis of aspkyncin using purified AacA demonstrated that the minimal NRPS module was sufficient to construct and cyclize the linear peptide into the cyclic product. The proposed cyclization mechanism, as illustrated in Figure 5, involves the nucleophilic attack of the secondary amine group of *N*-methyl-l-alanine on the thioester of *N*-methyl-l-alanine-l-Kyn-S-T2, resulting in the formation of aspkyncin.

By integrating the findings from both in vivo and in vitro experiments, a plausible biosynthetic pathway for aspkyncin was proposed. As illustrated in Figure 5, AacB mediates the synthesis of the unnatural amino acid *N*-methyl-l-alanine, IDO catalyzes the ring-opening oxidation of l-Trp to form *N-*formyl-l-Kyn, which is subsequently hydrolyzed to l-Kyn through an endogenous l-Kyn formamidase. The NRPS AacA then incorporates one molecule each of *N*-methyl-l-alanine and l-Kyn to assemble the dipeptide scaffold, which is further cyclized to form DKP spontaneously without the involvement of a release domain.

## 4. Discussion

Fungal DKPs are typically formed through the condensation of two natural amino acids. These compounds have garnered attention not only as challenging synthetic targets but also due to the diverse and intriguing biological activities exhibited by some of them [4]. The most abundant and structurally diverse DKP natural products are those based on the cyclo (l-Trp-l-Pro) core, which is derived from the condensation of tryptophan and proline residues [4]. Additionally, other proteinogenic or non-proteinogenic amino acids have been identified as participating in DKP synthesis. In this work, the aspkyncin we isolated and characterized represent the first fungal DKP containing a l-Kyn unit, thereby expanding the diversity of fungal DKPs.

In fungal genome sequencing projects, a great number of new NRPS genes have been identified, but the products of the NRPSs they encode have not been detected. This implies that many unknown NRPS products exhibiting potentially interesting properties still await discovery [26]. A minimal module for the incorporation of one amino acid into the growing peptide chain consists of an adenylation (A) domain, a thiolation (T) domain, and a condensation (C) domain. A typical linear DKP template has the domain organization A-T-C-A-T-TE/C_T_. The final step in DKP synthesis is the release of the covalently bound peptide from the synthetase. In bacteria, C-terminal thioesterase (TE) domains commonly release the final product. However, in fungal NRPSs, termination of non-ribosomal peptide synthesis may also be catalyzed by reductase (R) domains or C_T_ domains (Figure S7) [15,27,28]. For example, gliotoxin requires a couplet of condensation (C) and thiolation (T) domains in the NRPS GliP [1,29]. However, we found that the NRPS AacA with the minimal domain organization A-T-C-A-T can efficiently catalyze the synthesis of aspkyncin, indicating that the cyclization and release of DKP can be completed spontaneously. AacA is the simplest fungal bimodular NRPS that has been functionally verified so far.

The direct catalytic function of AacB in *N*-methylation of l-alanine is unexpected. To date, glycine *N*-methyltransferase (GNMT) is the only known methylase that catalyzes the *N_α_*-methylation of proteinogenic amino acids [30]. GNMT catalyzes the transfer of a methyl group from SAM to glycine, forming sarcosine and S-adenosylhomocysteine, thereby regulating methyl transfer reactions in the human body. *N*-methylations of the peptide backbone are typically catalyzed by integral methylation domains within NRPSs [8]. A prominent example is cyclosporine A, a cyclic undecapeptide that contains seven *N*-methylated amide bonds (Figure S8) [31]. Some non-proteinogenic amino acids are modified by methylation as building blocks. Standalone methyltransferases, such as the structurally characterized glycopeptide *N*-methyltransferase MtfA, are also known to modify NRPs after their assembly [32].

The discovery of AacB represents a novel *N*-methylation pathway for cyclic peptide compounds that occurs prior to NRP assembly. This simple modification of peptide backbones through *N*-methylation significantly enhances the pharmacokinetic properties of natural peptides [33,34]. Consequently, research on AacB holds substantial promise for future drug discovery initiatives and serves as a crucial element in the field of synthetic biology.

## 5. Conclusions

In this study, we identified a novel diketopiperazine alkaloid (aspkyncin) from *A. aculeatus* CRI323-04 through an integrated approach of genome mining and heterologous expression. A unique amino methyltransferase, AacB, was found to catalyze the *N*-methylation of l-alanine. To the best of our knowledge, this is the first reported instance of an l-alanine *N*-methyltransferase. We successfully reconstituted the activities of the bimodular NRPS AacA both in vivo using *A. nidulans* LO8030 and in vitro. AacA represents one of the minimal enzymes catalyzing DKP synthesis in fungi, comprising only five domains: A-T-C-A-T. Notably, it recognizes two non-natural amino acids simultaneously, a rare feature in DKP biosynthesis. Due to the important physiological functions of Kyn derivatives, aspkyncin can serve as a lead compound in drug discovery programs. Additionally, homologous gene clusters encode other tailoring enzymes, such as FAD-dependent oxidoreductases and P450 monooxygenases, providing a valuable resource for discovering novel l-Kyn-containing metabolites.

## Data Availability

The original contributions presented in this study are included in the article/Supplementary Materials. Further inquiries can be directed to the corresponding authors.

## Supplementary Materials

The following supporting information can be downloaded at https://www.mdpi.com/article/10.3390/jof11030171/s1: Figure S1: Schematic diagram of plasmids used in this study; Figure S2: The metabolic profile of the mutant strain with high expression of the transcription factor gene *aacF*; Figure S3: Structures of selected natural products derived from l-kynurenine; Figure S4: Amino acid sequence alignment in different A domains; Figure S5: In vivo feeding assay of AacA; Figure S6: SDS-PAGE analyses of the purified proteins GST-tag-AacB (~66.3 kDa); Figure S7: Release mechanism of fungal bimodule NRPS; Figure S8: Structure of natural cyclic peptides containing *N*-methylation; Figure S9: HR-MS spectrum (positive ionization) of aspkyncin; Figure S10: ^1^H NMR spectrum of aspkyncin in DMSO-*d*_6_; Figure S11. Expansion of the 8.5–6.0 ppm region of ^1^H NMR spectrum of aspkyncin in DMSO-*d_6_*; Figure S12. Expansion of the 4.5–1.0 ppm region of ^1^H NMR spectrum of aspkyncin in DMSO-*d_6_*; Figure S13: ^13^C NMR spectrum of aspkyncin in DMSO-*d*_6_; Figure S14. Expansion of the 200–110 ppm region of ^13^C NMR spectrum of aspkyncin in DMSO-*d_6_*; Figure S15. Expansion of the 60–10 ppm region of ^13^C NMR spectrum of aspkyncin in DMSO- *d_6_;* Figure S16: DEPT 135° spectrum of aspkyncin in DMSO-*d*_6_; Figure S17: ^1^H-^13^C HSQC spectrum of aspkyncin in *DMSO-d*_6_; Figure S18. Expansion of the 8.5–6.0 ppm (^1^H) and 200–100 ppm (^13^C) region of ^1^H-^13^C HSQC spectrum of aspkyncin in DMSO-*d_6_*; Figure S19. Expansion of the 4.5–1.0 ppm (1H) and 100-10 ppm (^13^C) region of ^1^H-^13^C HSQC spectrum of aspkyncin in DMSO-*d_6_*; Figure S20: ^1^H-^13^C HMBC spectrum of aspkyncin in DMSO-*d*_6_; Figure S21. Expansion of the 8.5–6.0 ppm (^1^H) and 210-50 ppm (^13^C) region of ^1^H-^13^C HMBC spectrum of aspkyncin in DMSO-*d_6_*; Figure S22. Expansion of the 5.0–1.0 ppm (^1^H) and 210-10 ppm (^13^C) region of ^1^H-^13^C HMBC spectrum of aspkyncin in DMSO-*d_6_*; Figure S23: ^1^H-^15^N HMBC spectrum of aspkyncin in DMSO-*d*_6_; Figure S24: ^1^H-^1^H COSY spectrum of aspkyncin in DMSO-*d*_6_; Figure S25. Expansion of the 8.5–3.5 ppm region of ^1^H-^1^H COSY spectrum of aspkyncin in DMSO-*d_6_*; Figure S26. Expansion of the 3.5–1.0 ppm region of ^1^H-^1^H COSY spectrum of aspkyncin in DMSO-*d_6_*; Figure S27: ^1^H-^1^H NOESY spectrum of aspkyncin in DMSO-*d*_6_; Figure S28. Expansion of the 8.5–3.5 ppm region of ^1^H-^1^H NOESY spectrum of aspkyncin in DMSO-*d*_6_; Figure S29. Expansion of the 3.5–1.0 ppm region of ^1^H-^1^H NOESY spectrum of aspkyncin in DMSO-*d*_6_; Table S1: Bioinformatics analysis of the *aac* gene cluster; Table S2: Primers used in this study; Table S3: Plasmids used in this study; Table S4: NMR data of aspkyncin; Table S5: The nucleotide sequences of *aacA* and *aacB*.
