# Supplementary material for: Biosynthesis of a Novel Diketopiperazine Aspkyncin Incorporating a Kynurenine Unit from Aspergillus aculeatus
Source: J Fungi (Basel). 2025 Feb 20;11(3):171. doi: 10.3390/jof11030171 (PMC11942691; doi:10.3390/jof11030171)
Supplement: Supplementary file 1 [file jof-11-00171-s001.zip › jof-3469910-supplementary.pdf]

Supporting Information

Biosynthesis of a Novel Diketopiperazine Aspkyncin Incorporating a Kynurenine Unit from *Aspergillus aculeatus* CRI323-04

Dekun Kong [1,*], Xin Wang [1] and Li Liu [2,*]

[1] College of Pharmaceutical Sciences, Southwest University, Chongqing 400715, P. R. China
[2] Laboratory of Biochemistry and Molecular Biology，Lab Teaching and Management Center，Chongqing Medical University，Chongqing 400016
* Correspondence: kongdekun@swu.edu.cn (D.K.K); liuli102687@cqmu.edu.cn (L.L.)

Supplementart tables

Table S1. Bioinformatics analysis of the *aac* gene cluster.

Genes	Proteins	Size (bp/aa)	Putative Function
aacA	AacA	1572/523	NRPS
aacB	AacB	5265/1754	Methyltransferase
aacC	AacC	1068/355	P450
aacD	AacD	1239/412	L-saccharopine oxidase
aacE	AacE	1371/456	BTB/POZ domain-containing protein
aacF	AacF	1005/334	Zinc finger-containing protein
aacG	AacG	795/264	Carboxylesterase

Table S2. Primers used in this study.

Primer name	Primer sequence (5′→3′)
pANR-aacA_F1	ctaaccattaccccgccacatagacacatctaaacaatgacaagagtcgacacttgc
pANR-aacA_F2	gatgtcgtcttcggcctcac
pANR-aacA_R1	gattgccgggccagtcatg
pANR-aacA_R2	gtagacctatcaatgatgatgatgatgatgatgatgcggattaccacgacgcatc
pANR-aacA_F3	ggtaatccgcatcatcatcatcatcatcatcattgataggtctacttggctggatg
pANR-aacA_R3	ctaaagggtatcatcgaaagggagtcatccaatttaaatcacagcatcggtccaatag
pANP-aacC_F1	cttctctgaacaataaaccccacagaaggcatttatggtgtcacaactttggaagac
pANP-aacC_R1	gatgagacccaacaaccatgataccaggggatttaaatcactgtcctggctgattgtg
pANU-aacBDG_F1	gagcctgagcttcatccccagcatcattacacctcagcaatgcgctcgttaagcactg
pANU-aacBDG_R1	gtctctcccgtcacccaaatcaattcaccggagtttggaccgatgctgtgaaatgag

Primer name	Primer sequence (5′→3′)
pANU-aacBDG_F2	ctaaccattaccccgccacatagacacatctaaacaatgtctataaccaccgtctgc
pANU-aacBDG_R2	gttatatcatttatagctcgttcggcacctttaatcgacgcatgctgtgctacaaatg
pANU-aacBDG_F3	ctctgaacaataaaccccacagaaggcatttatggaccacaagaataagagctacaac
pANU-aacBDG_R3	cagtggaggacatacccgtaattttctgggcatttaaatggcgggtagaggagaagg
gpdA-F	actccggtgaattgatttggg
gpdA-R	tgtttagatgtgtctatgtggc
AmyB-F	gattaaaggtgccgaacgagc
AmyB-R	aaatgccttctgtggggtttattg
glaA-F	cctgatcttccgaactggtcg
glaA-R	tgctgaggtgtaatgatgctg
pANR-aacAB_F1	ctaaccattaccccgccacatagacacatctaaacaatgacaagagtcgacacttgc
pANR-aacAB_F2	gatgtcgtcttcggcctcac
pANR-aacAB_R1	gattgccgggccagtcatg
pANR-aacAB_R2	gaccagttcggaagatcaggaaatcacagcatcggtccaatagacatagaaatactcg
pANR-aacAB_F3	ctgagcttcatccccagcatcattacacctcagcaatgtctataaccaccgtctgc
pANR-aacAB_R3	ctaaagggtatcatcgaaagggagtcatccaatttgacgcatgctgtgctacaaatg
PGEX-aacB_F1	ccggaattcatgtctataaccaccgtctgc
PGEX-aacB_R1	aaggaaaaaagcggccgcctatgaagacaaaagatagaatcc

Table S3. Plasmids used in this study.

**Name**	**Description**	**Enzyme site**	**Funtion**
**pIM2513**	*aacA* in PANR	*Bam*HI	*A. nidulans* heterologous expression and protein
**pIM2516**	*aacC* in PANP	*Bam*HI	*A. nidulans* heterologous expression
**pIM2522**	*aacB*+*aacD*+*aacG* in PANU	*Not*I	*A. nidulans* heterologous expression

**pIM2515**	*aacA*+*aacB* in PANR	*Bam*HI	*A. nidulans* heterologous expression
**pIM2562**	*aacB* in pGEX-4T-1	*Eco*RI/*Not*I	protein expression in *E. coli*

Table S4. NMR data of aspkyncin.

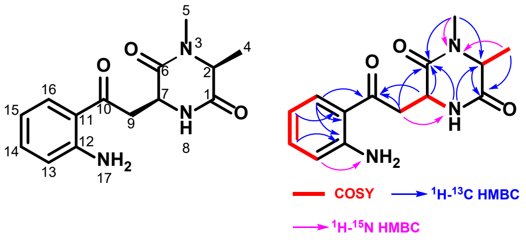

(400 MHz for ^{1}H NMR, 100 MHz for ^{13}C NMR)

No.	δ_H, mult (J in Hz)	δ_C, type	δ_N, type	^{1}H-^{13}C HMBC	^{1}H-^{15}N HMBC	COSY
1		167.4, C				
2	3.96, q (7.0)	56.7, CH		C1, C4		H4
3			113.1, N			
4	1.52, d (7.0)	17.5, CH_3		C1, C2	N3	H2
5	2.85, s	31.5, CH_3		C2, C6	N3	
6		165.5, C				
7	4.28, m	51.0, CH		C6, C10		H8, H9
8	8.02, s		116.7, NH	C1, C2		H7
9	3.40, d (5.0)	42.5, CH_2		C6, C7, C10	N8	H7
10		198.1, C				
11		116.3, C				
12		151.2, C				
13	6.76, d (8.3)	117.0, CH		C15	N17	H14
14	7.25, m	134.4, CH		C12, C16		H13, H15
15	6.54, t (7.2)	114.5, CH		C11, C14, C16		H14, H16
16	7.73, d (7.3)	131.2, CH		C10, C11, C12, C14		H15
17	7.20, s		72.9, NH_2	C13		

Table S5. The nucleotide sequences of *aacA* and *aasB*.

Gene name	Nucleotide sequence (5′→3′)
aacA	atgacaagagtcgacacttgcgaagatgccacccctcaaaagttcacccattccacaaagacaaccaacaataaccgag<br>aattgctacgagaagtgggtgacaccgagctcgaccagctgcagctgcagcaattaaactccttcaaccccccaacacac<br>cagcccgccaatgtctgcatacatgaactgtttcacagtcaggcagttgcacatcctgaacgcgaggccgtatccgcttgg<br>aatggacagtttacttttgccgctctcgatcgcctgtcgtcctgcctcgcgtcacacatactgaatctcggagtcgcaatcga<br>ggcgttcgtcccggtgtgctccgagaagtcatgctggtatcccgtggcactgctgggggtcttgaaagccggggctgcctt<br>tgtgccgttggatccatcccatcccacccagcgcttagaggagacctaccgcgccgtcgcagcgaaggtgatcatcgcc<br>accaaggccacggccgagaaggtcaagtttgctgacaaggttgtcgtggttgatgatgacgatggcgatggtgatgtcac<br>tgctcctggcagaggggcaaccacgctgcacccagagccagaggttccctgcaccagtactgcctacgcagtcttcacg<br>tccgggacttccggaaaaccgaagggggtggttatagagcatcggtctctatcgactagcgttctggctaacagtgccgc<br>gctgaatatccatgacaagacccgtgtcttccagtttgcgtcccatgcgttcgacgcctcgctgttggatatcttcgcgccgtt<br>ggtcatgggcggttgtgtgtgcatcccgtcagaggctgagcggaagaacaatctggcagaggcatgtcgccggcttaact<br>gtacttggtccttcttgactccctcgctgacaagggtgttggagccggaggatttatgcaccctgcagaccctggtgattgg<br>aggcgaggctctgcgagaaggggacattgagaaatggttgcctcgtatgcagtgcatcagcggttatggtcccaccgaat<br>gcacgatcggctgcatggctacgacgctccaaccaggcgtccggcctgacccgtctgatctggggcaaggggttggagt<br>caattgctgggtcgttgaccccgacaaccatgacagattgagtccgcctgggaaggttggagaactgctgctggaggga<br>cccatggtcgcccgcggctacttcaatgaccccaaacgaacggccgaagtgttcatcagcccccccggtgtggcacaaaa<br>tgttgagattccgaggtgatcggatgtacaagacaggggatctggtggttttcgacgccgtgaaaagcgcgatccgttacg<br>ttgccagaaaggatacgcagatcaagctccacggccagcgtctggacacgggagaggttgagcatcatatccgatcgat<br>gcttccaagcgccgtggatgttgtggtagatatgattcatcttgcagatgagacttcagcctcaatcctcgttgcatttcttgttc<br>tcaaagcagatagtacaacaacgcacggcaccaaggcagaatggcttgaggaaccgacggatgacctgagcactaacg<br>tggagatgcttaaagccaaattgtccgatctggttcctgagtacatgatccccagcgcatttttacctctagtgtcaatgcctct<br>cacgaacaacggaaagggtgaccggaaaatgcttcgcaatctggctgcgaccttgacccgacatgatctccgcaggtat<br>aacggaaactatgctgtcgagaacagcaggccttccagaccagttgagcgcaagctccagatgctgtggtcgaaagtact<br>cgagattccagtggatgagattggtcttcaagatggcttctttgctcttggtgggacttctgtggcagccatgaaactggccg<br>gtctagcgaggaaagaaggcctcgaattagacatagccgacttatatggtcaccctgccctcaaagacatcatgacccga<br>gtccgcacgatctcgagagatagtgcaatccagccgttctcgctggttcctgccagtgaagacttcaagcagatcgttaca

gagaccatgaagcagtgcgggctcagccacgaaacagaaatcgaggacatgtatccttgtacaccgttgcaggaagga
cttatatctctcgccgctaaaagaccgggctcctatattggcgtgtttcggtacgaaatgcctgcgaaggtgaatactcaccg
gttccagtccgcatgggatgcgttagtcgacgctcacccgatcttgaggacccgatttatccaaaccagcagtggcaagat
ctaccaggcagtaattcggaggtcagtcgcgttagatgtgagcacgctgaacagagagggaatcagggcagcaaaaag
ttcgctttcctgcgagctcggacagtcctggatccgtgcacatcttcacaatacagcatctactttacagctcccgaccttcac
actgactgtgcatcacgctttgtccgatggctggggccttcccgtactccttcgggacctcgaagccgcctacaacggaca
tacactggaagagaggccgttcaatcctttgatcgagtacatcgggcgcacgactaccgagcgagaggcgtattggcaa
ggccgctttactgatatgcagagtgccaggttcccttcgctaccctccccagcgtatgtcccaaaggctacggaaatgcatc
caatctgtattcccgtggaccatcgggttgcaagtgagtacacacttgctgacaaactcaagctagcatggggatcatcct
ttccctttatacggagaccgccgatgtcgtcttcggcctcactgttagcggacgtggcgcccctgtgctgggcgtcgagga
catgactggcccggcaatcgcaaccatccctttgagactgaatctcgatccagagatgaccgtgaagaagagtcttcagc
ggttgcgggagttctgtgttgccgcgatgccatacgagcacatagggcttcaaaaccttcgacgccttggtcagggaccta
gcaaagcttgtgatttccagagccatgttgtcatccaacccgaggagactggcagtgattggatgttcgcaaggaagcag
gactattcggaaactggagcattcagttcctacgccattaccttgatctgtcagcagaaaccgggctcaatagaaatcgagg
cgacattcgatccgcacgttgtccccagggtgcaaatggaccgattgctgaagcagcttcgacatatcatgcaggttctgat
ttctggatccgaagaagcttgtctgtgtgacctggacacgatctgtcgtgaagattggtcacgattgaaacaatggaatacc
gcgcttcccgaggcggtggatatctgtgctcacgagtggatccgaaagcgaagtctcgcgcagcccaacgctccagctg
tgtgcgcttgggatggtattttcacttacgaggagatggagcagacttcatttgagatggctgcctatctccgagaacaagg
cgtaggccctaatgtgtttatccctctctttttcgaaaagtcatgctggaccagcatcgcaatgcttgcagtgatgaaggccg
gaggcgcatttattctgctagacgactctcatccagtacaacgactgcgtggaatatgcgatgatgcagaggcgcctttcat
tatcacgtcggagaagaatgcacctcgtgcaggtcaaattgcaaattcttccatcatactgggtgattcgcaccgatcgtgg
ccacagagtcagacaaaacccgcgactcccatgtcacaggtcaaccccagagatgcgctctatgcagtcttcacctcggg
ctcgacaggaaagcctaaaggcgccgttgtgtcgcatcagtcgtggtgcacaagcgccaaagccaacagcgttgcattgt
cattgcgtcctacttcccgcgtctttcagttcgccgcctacgcttttgatatcagcatcgcagatcatttgctgactttcgtggct
ggcggttgtgtctgtattccttctgccgaagatcgagaaggaggccttgctcagatcatcagtagactcgaggccaattgg
gcatgcctcacgccgtctgtgtcgcgcattatcgagcctcaaaaagttccaacactggagacgctggtactagcaggaga
gcctattgcaccggaggatatctcgatgtggtcaccagcagttcacctgttgaatctatatgggcccgcggagtgtgccatc
cttaccactctgaatcaagaggtcaacaactcaagtgatccgaacaacgtcgggcctccgaccagcgcagtgtgctgggt

	tgtggatacaagaagcgtcgacaaactagtacctatcggaacagtgggagagctcgtggtcgaaagccctattgtaggct<br>acgggtacctcaacaacccgatcaagacggctgcctcctttatcgccccagaggcgcacccaccgtggctgcagcgatt<br>ccgacgcacagaaaccgcccgttcgcgcctctaccgcacgggtgatctggtccagtacgcagaagacggaaccctgag<br>gttcatcggccgcaaagacacgcagatcaagttgcgcggacaacgcatcgagctgggcgaggtcgaataccacctgcg<br>ccgcagctttcctgatgccgccgaggtcgtggccgaggtcgtcgtcccgaacgatgatagatcacatagccgaaggcag<br>ccgaccctgacggccttcatccgtccacgggagcagactaccccccataaatcaggcccctcgcttctccaagcgcaaat<br>tgccacagcgctgatagccctgcgcgcctcactgcccacatacatgatcccgaccgcattcctgtgcgttgattcatttccc<br>ctgagcaagtcccgcaagctggaccgcggccagctccgcagcatggccgctgcgctgccccgagaggcatacctcgat<br>gcacccaccctgcagaagcgggagccgtccagcccagcggaggagatgttgcatcggctgtttgcggacactctgaag<br>atcgaccccgcggagttcgggatcgaccatgacttctttgggctgggcggcgactccatcctggctatgggtctggtggc<br>gcgggcgcgagcgcgttgcttggatttcaccgtggcggatttgtttgctcatccgacggtgggcgcgcttgctgggcattt<br>gatgcgtcgtggtaatccgtga
aacB	atgtctataaccaccgtctgcgaagttacttggccatgctcggcactatcgctcgatacgcgggctcccaaggaaccctttc<br>ttcgcgacattcgaaagtccttggagcttcttgacctgaagtcatgcgttatatcgggtctgttgcagccgcgcaaggcgatt<br>ccgtcacttctcctctgggacgagcaggggctcaaaaatttcaatgaatggaccaactgtcctcactactatcccaagtcaa<br>aggagttggagatcctcgagacgcacaaagacaagatggcaggcgctctacctgatcggtctgcgttagtcgagctagg<br>gtgtgggtgcgttcgcccgctttcaagatcccacgttcatgaatcatactcagtgcctgactccatcctagaaacgttcacaa<br>gaccgcaatcatactctccgccctcgctggacaaggcaaggaggtccaatactatgccctggacgtctcagaatcagcgt<br>tgcgcaccagcctgcaggctctgcaagcagagttcaagcacttcccgaacatcagtatctccggtctgctcggcacatatg<br>acgactgcgtagactggatcgccaacaacaccgccagtctccacacacgctcagtcacctttctctgggtcggcaatagtg<br>tggcaaatctctccaagaccgaagcaggcgaccttatgggccagttccgccaagcctgcgccgttgcaggaatgcactg<br>ccatttcttggtctctgcggacgcttgtgccgacgagagcaagctactcaaagcctacaatcccgaaggggggtttgtcgtcc<br>ctcttcctccgctatggcctacttcacgttaacaagctgctccgggcggatctgttcaacgacgtggtttggaactgcctcat<br>cgagtacgatcgggaggagaatgagattctgactttctattcccctgaaagcgatgtcacattgtcgagcggcgccacgtc<br>agtgacggtacatcacggggagaagatttatttcttccgtagtggtaaatggaatgaagagcaaatgagcagtattgcaca<br>gcagggcgggttccaactttcgcgggtatggagagacgcaaaacaggagtatggtaagctgagcccaaaggtaaattttc<br>aaggtggatggtgtgcagctactgacgaatttcaggattctatcttttgtcttcatag

Supporting figures

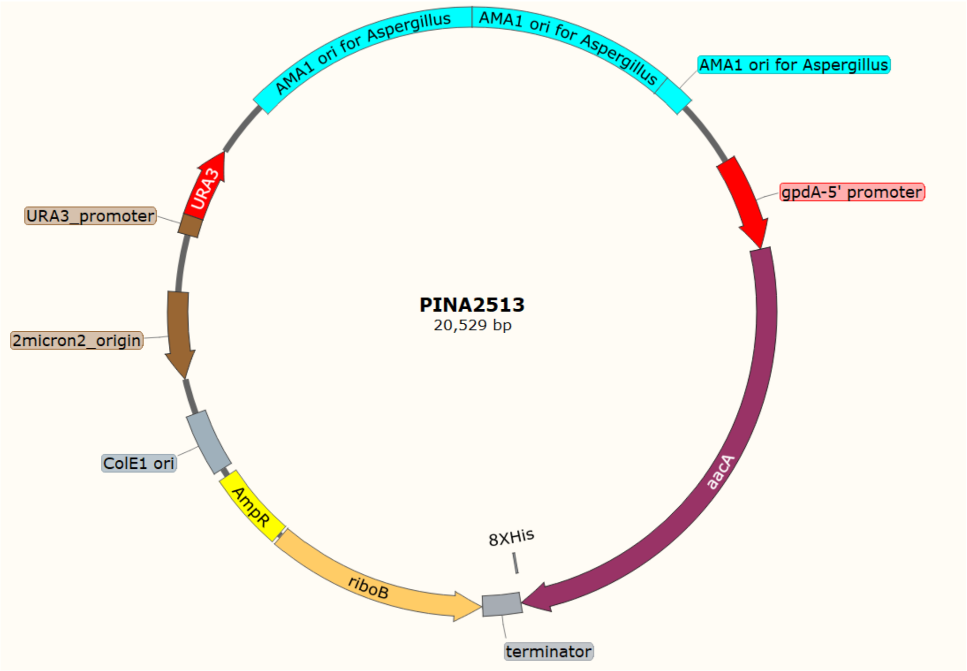

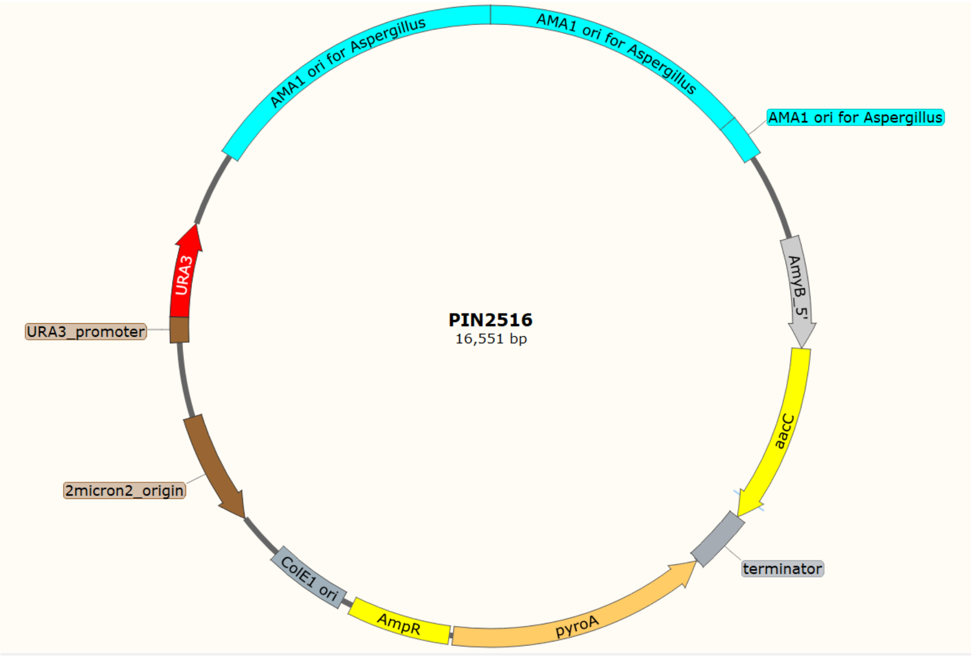

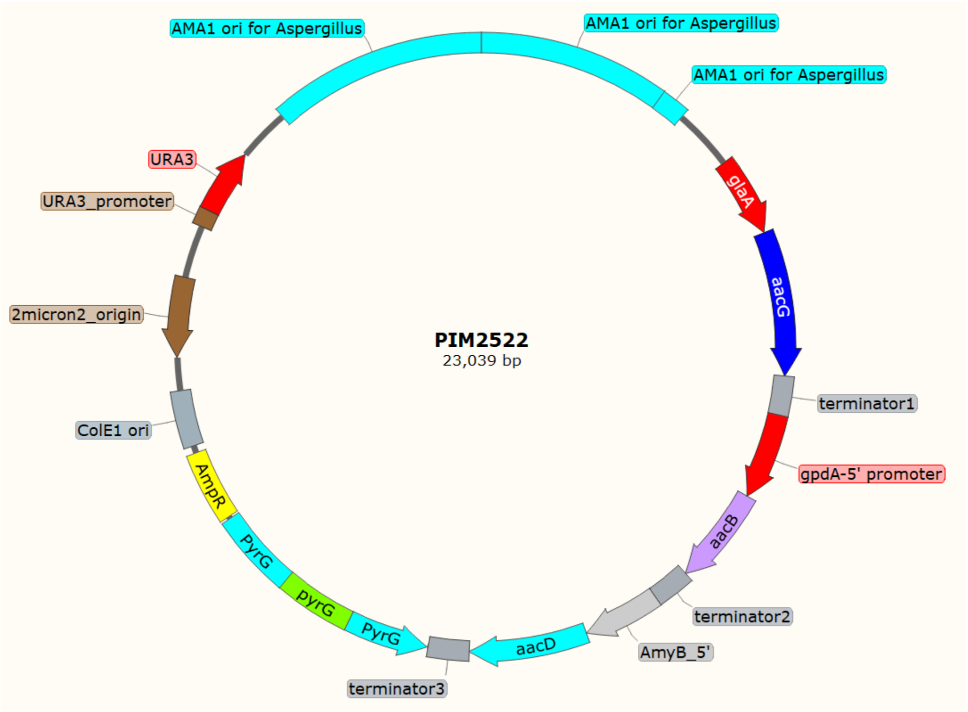
AMA1 ori for Aspergillus
AMA1 ori for Aspergillus
AMA1 ori for Aspergillus
URA3
URA3_promoter
2micron2_origin
ColE1 ori
AmpR
PyrG
pyrG
PyrG
terminator3
aacD
AmyB_5'
terminator2
aacB
gpdA-5' promoter
terminator1
aacG
glaA
PIM2522
23,039 bp

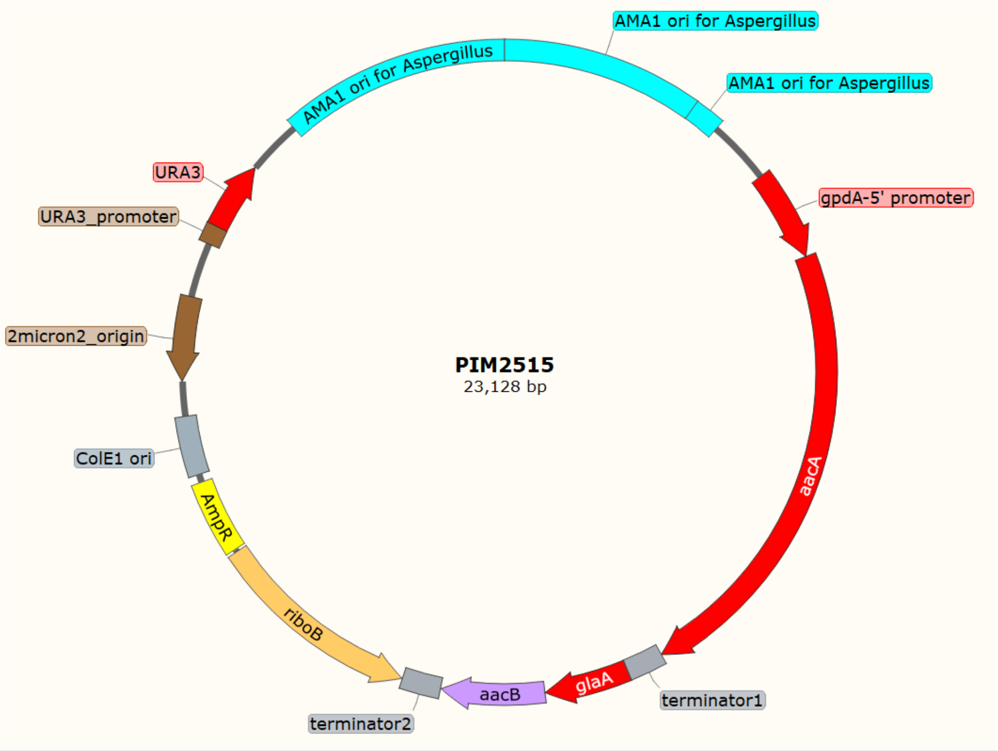
AMA1 ori for Aspergillus
AMA1 ori for Aspergillus
AMA1 ori for Aspergillus
URA3
URA3_promoter
2micron2_origin
ColE1 ori
AmpR
riboB
terminator2
aacB
glaA
terminator1
aacA
gpdA-5' promoter
PIM2515
23,128 bp

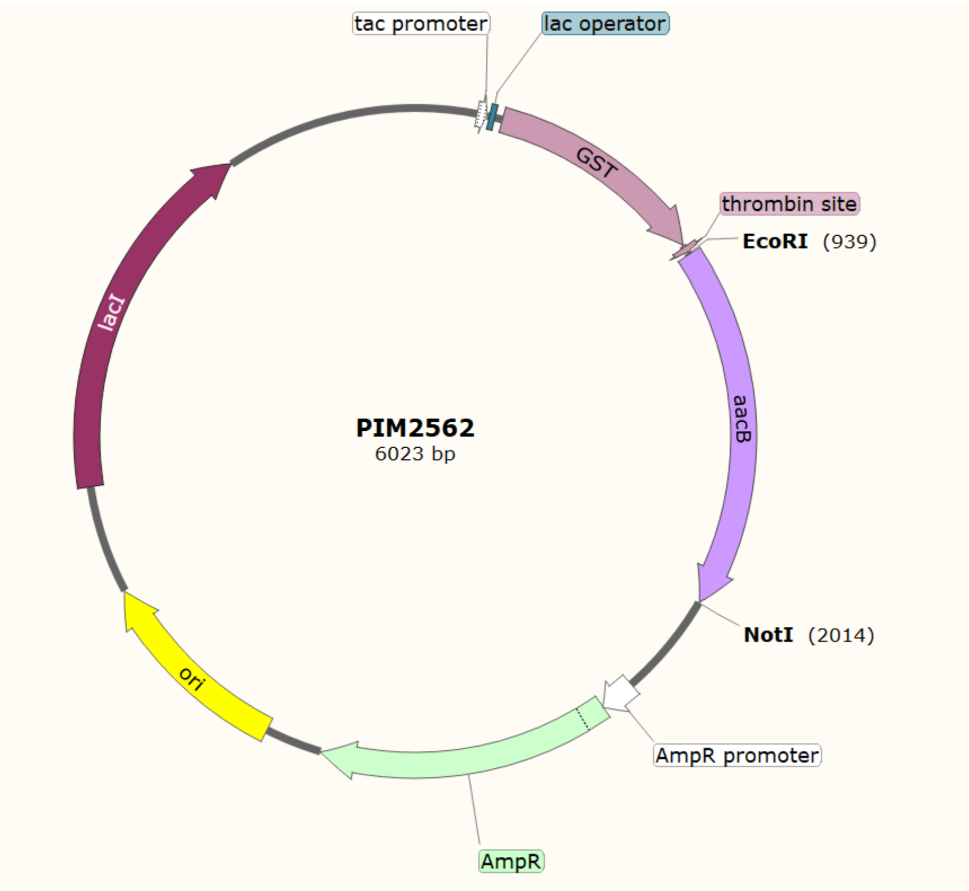

Figure S1. Schematic diagram of plasmids used in this study

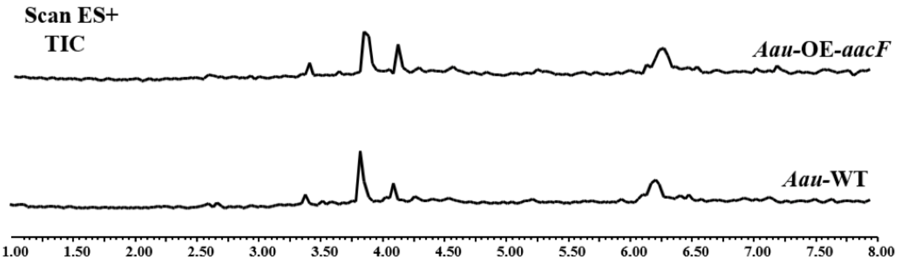

Figure S2. The metabolic profile of the mutant strain with high expression of the transcription factor gene *aacF*.

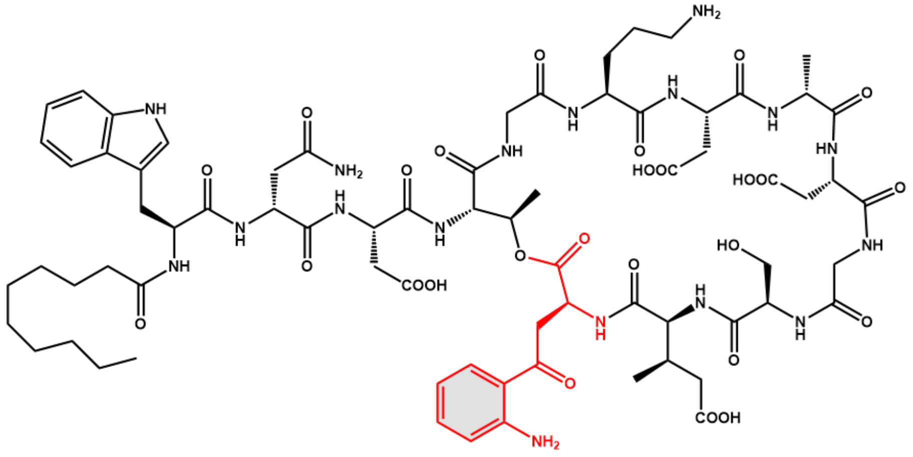

daptomycin

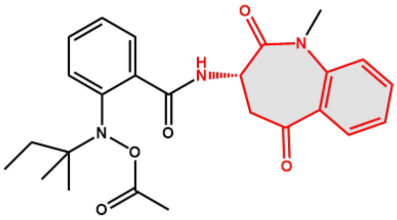

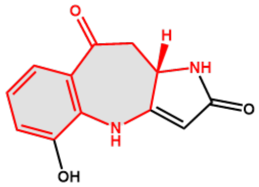

nanangelenin A aspcandine

Figure S3. Structures of selected natural products derived from L-kynurenine. The substructures derived from L-kynurenine are shown in red.

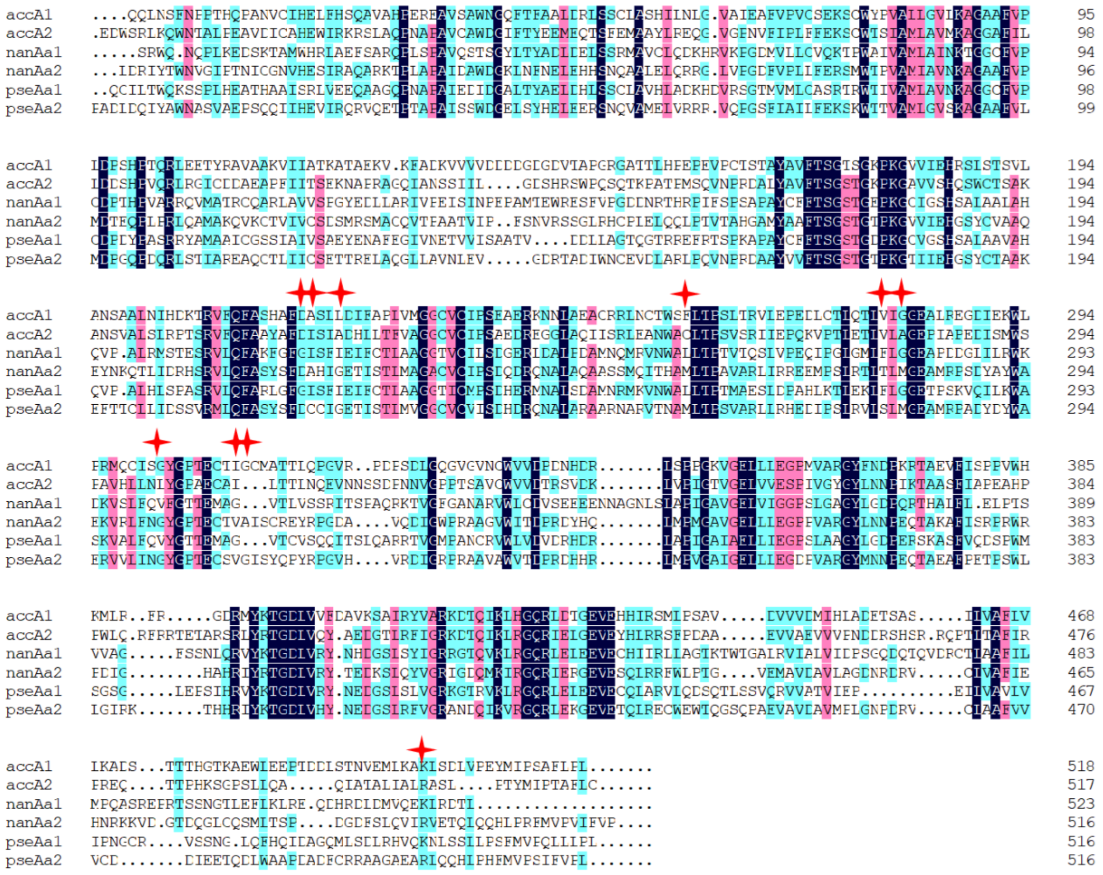

Figure S4. Amino acid sequence alignment in different A domains.

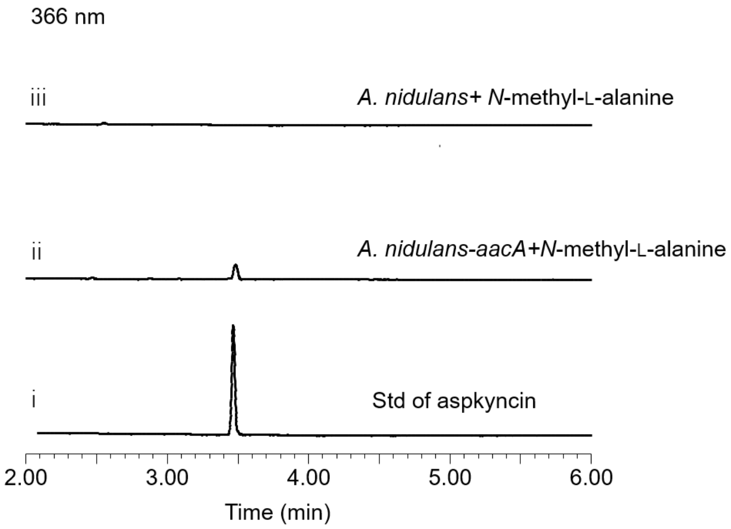

Figure S5. *In vivo* feeding assay of AacA.

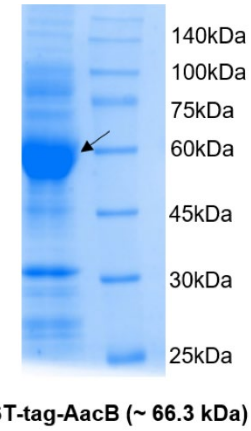

Figure S6. SDS-PAGE analyses of the purified protein GST-tag-AacB (~ 66.3 kDa).

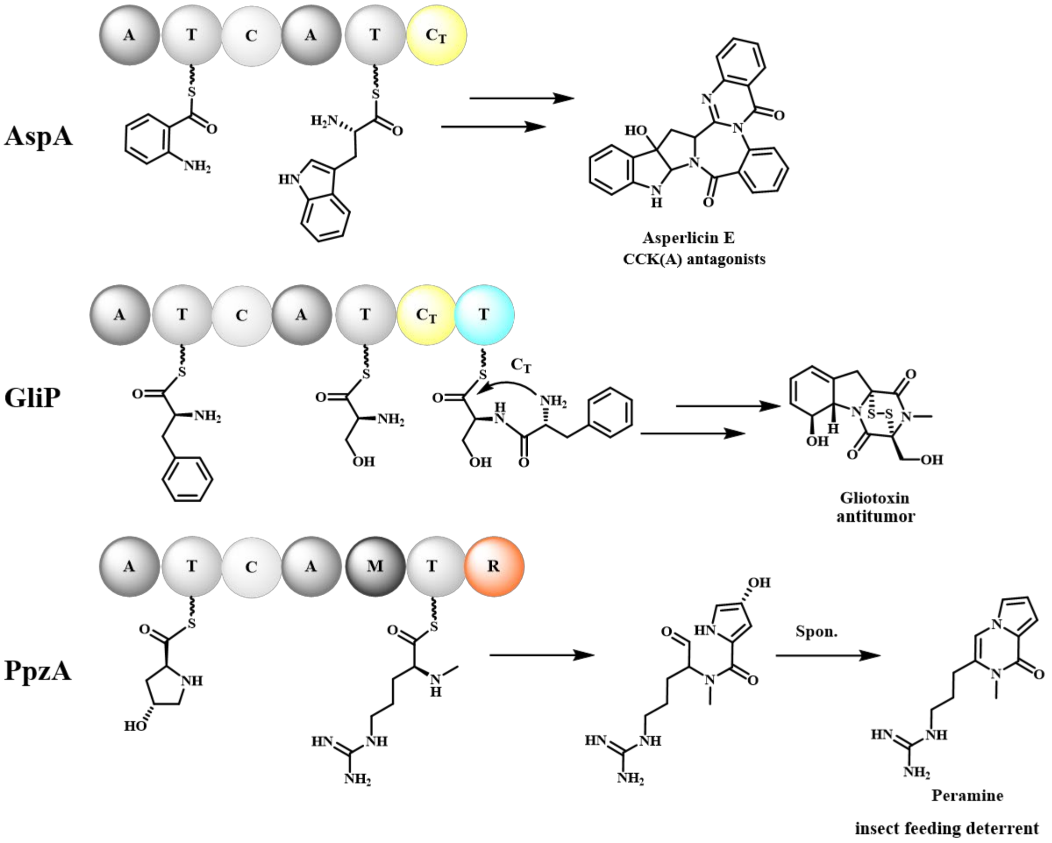

Figure S7. Release mechanisms of fungal bimodule NRPS.

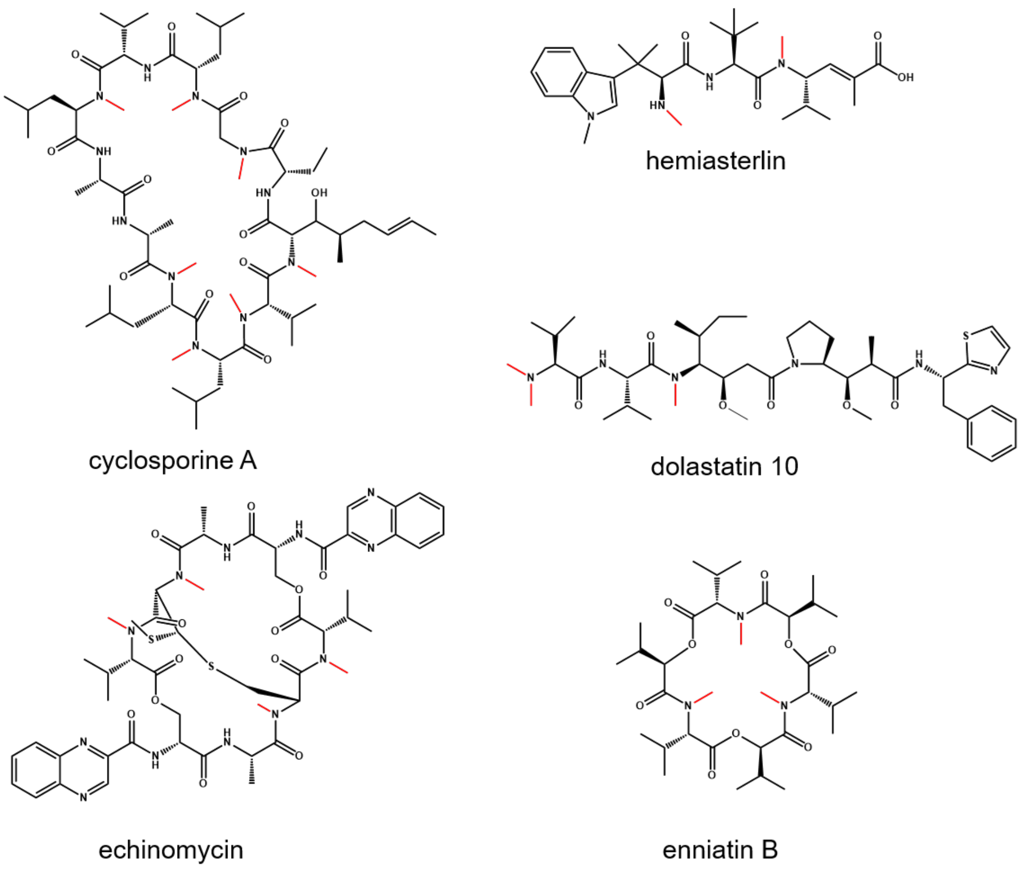

Figure S8. Structure of natural cyclic peptides containing *N*-methylation.

Mass Spectrum SmartFormula Report

Analysis Info

Analysis Name	D:\Data\2020\Zhouchenghe\20210106\WX-275.d	Acquisition Date	1/6/2021 11:12:58 AM
Method	DirectInfusion - MS - positive.m	Operator	Demo User
Sample Name		Instrument	impact II 1825265.10221
Comment			

Acquisition Parameter

Source Type	ESI	Ion Polarity	Positive	Set Nebulizer	0.4 Bar
Focus	Not active	Set Capillary	4500 V	Set Dry Heater	180 °C
Scan Begin	50 m/z	Set End Plate Offset	-500 V	Set Dry Gas	4.0 l/min
Scan End	1300 m/z	Set Charging Voltage	2000 V	Set Divert Valve	Source
		Set Corona	0 nA	Set APCI Heater	0 °C

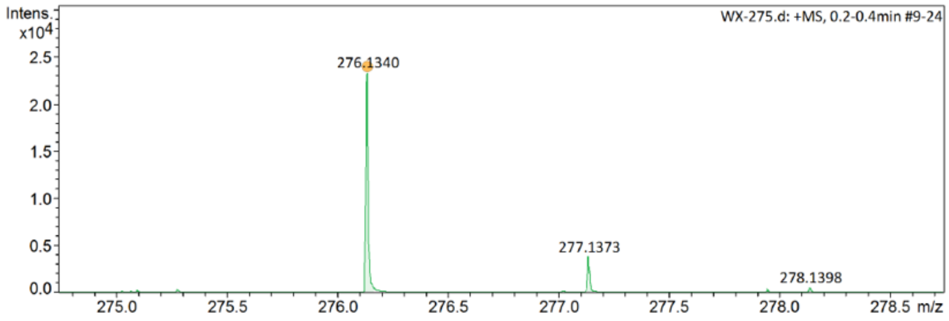

Meas. m/z	#	Ion Formula	Sum Formula	m/z	Adduct	err [ppm]	z	mSigma	Score	rdb	N-Rule	e⁻ Conf
276.1340	1	C14H18N3O3	C14H17N3O3	276.1343	M+H	0.8	1+	2.0	100.00	8.0	ok	even

Figure S9. HR-MS spectrum (positive ionization) of aspkyncin.

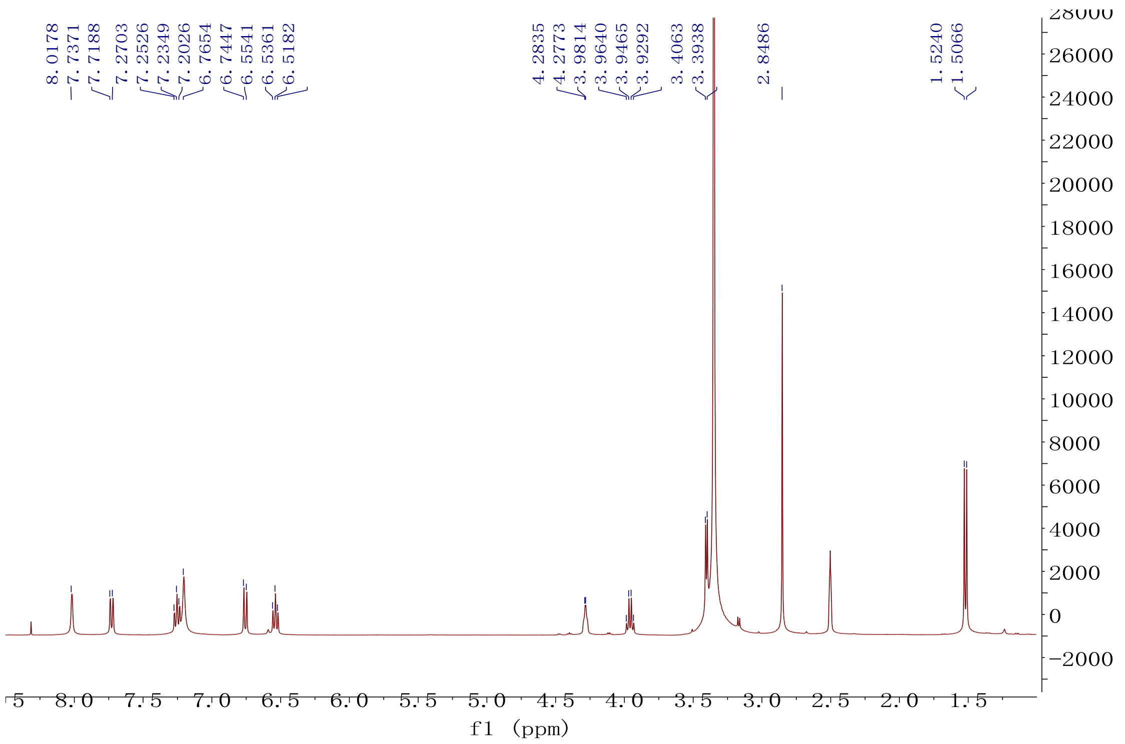

Figure S10. ^{1}H NMR spectrum of aspkyncin in DMSO-d_6.

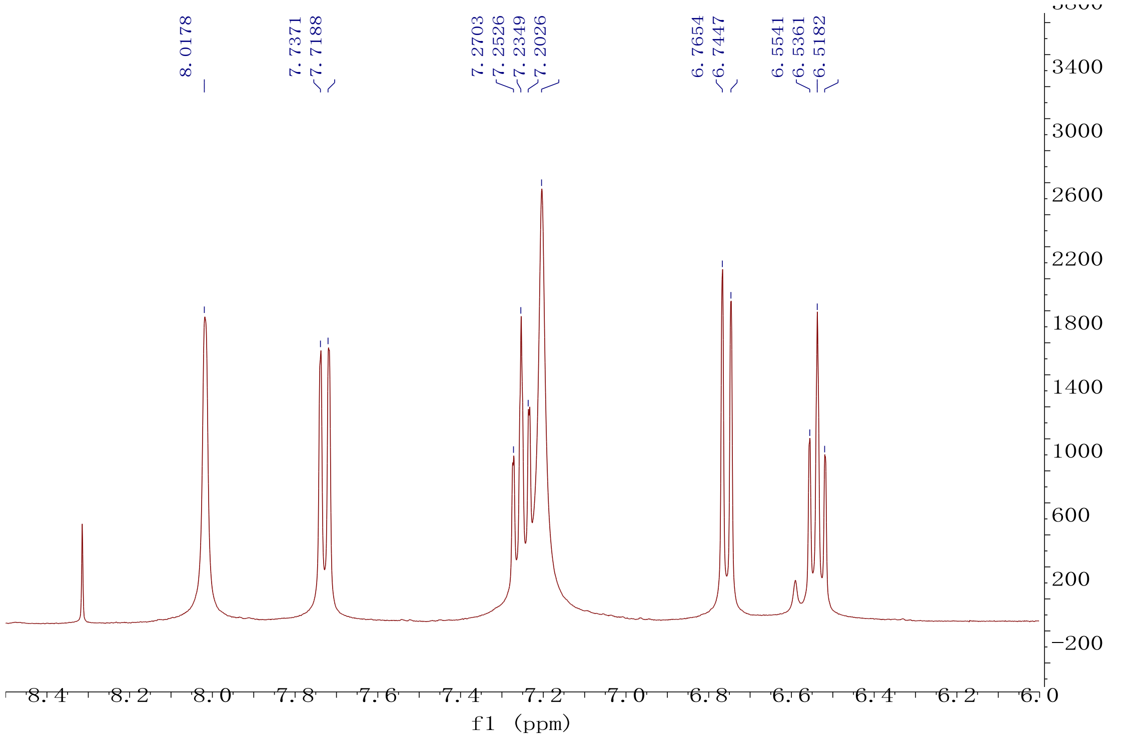

Figure S11. Expansion of the 8.5-6.0 ppm region of ^{1}H NMR spectrum of aspkyncin in DMSO-d_6.

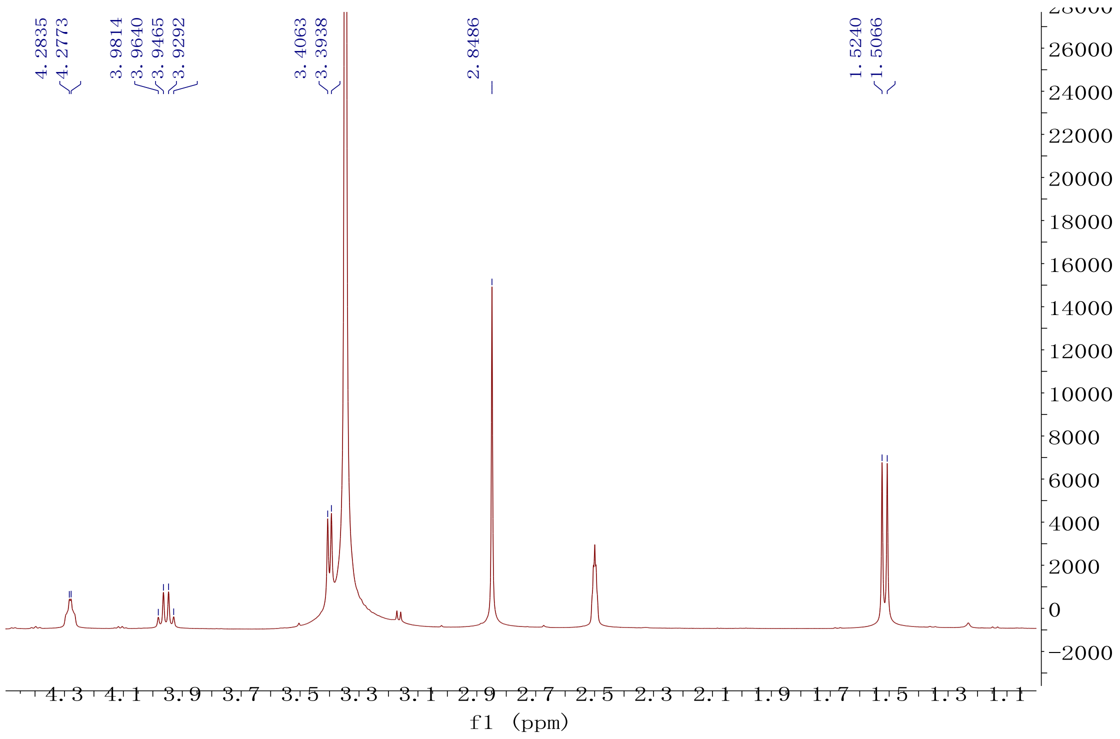

Figure S12. Expansion of the 4.5-1.0 ppm region of ^{1}H NMR spectrum of aspkyncin in DMSO-d_6.

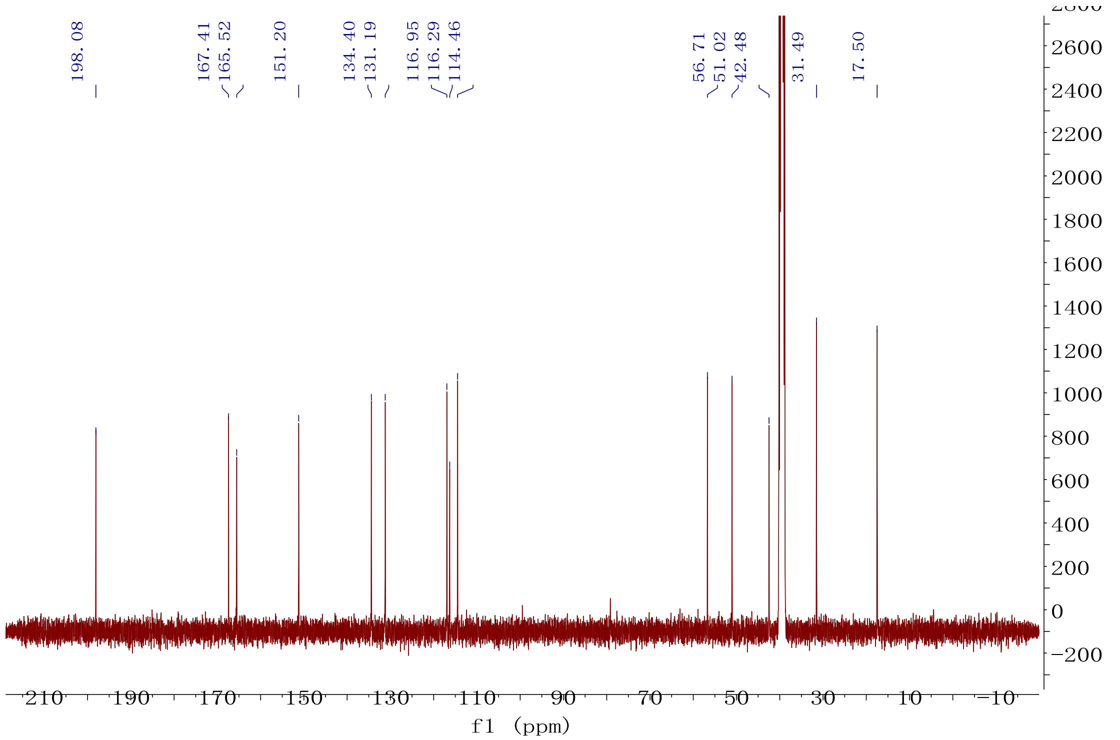

Figure S13. ^{13}C NMR spectrum of aspkyncin in DMSO-d_6.

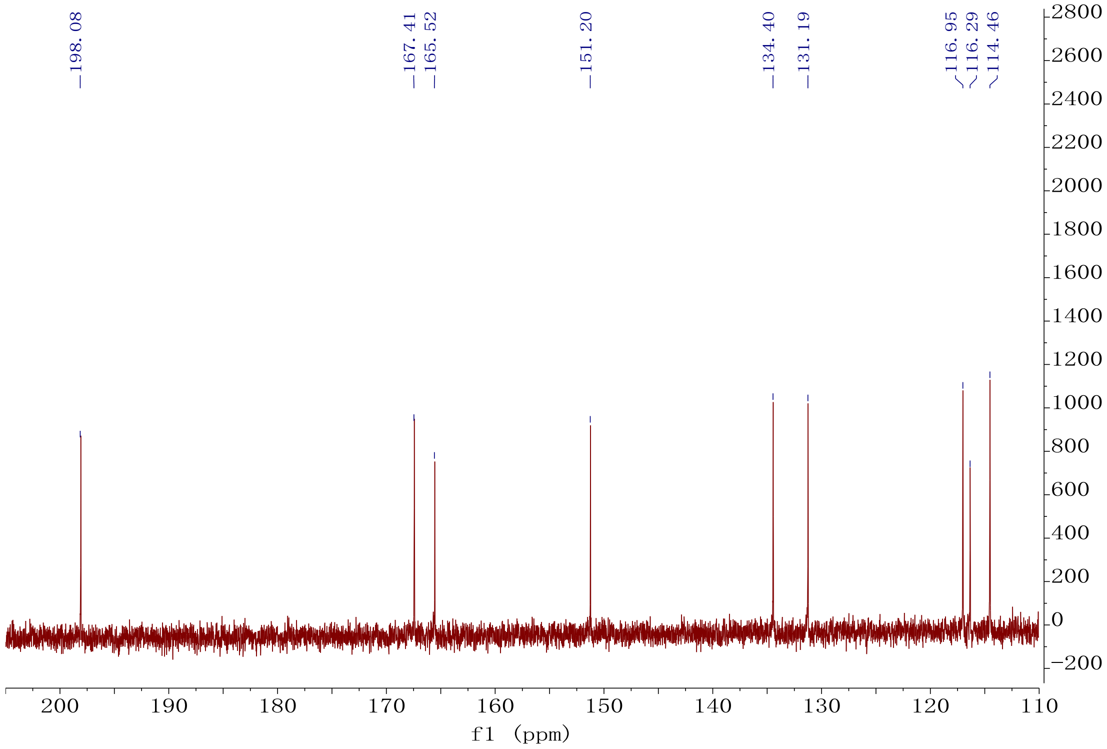

Figure S14. Expansion of the 200-110 ppm region of ^{13}C NMR spectrum of aspkyncin in DMSO-d_6.

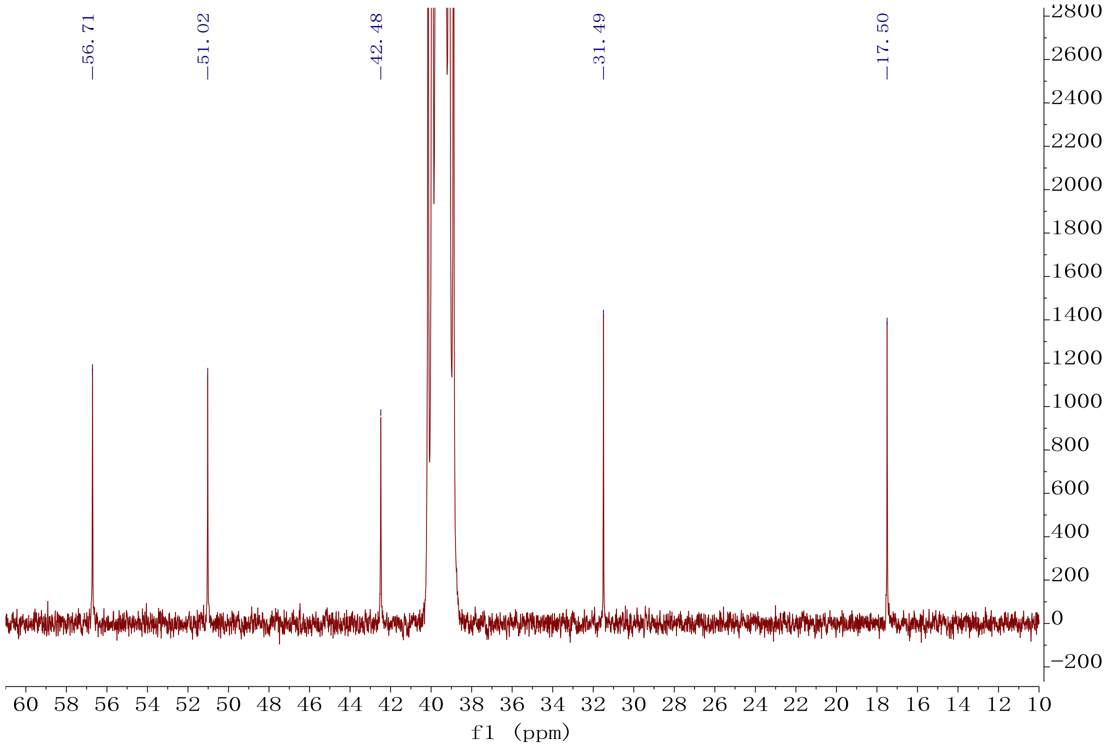

Figure S15. Expansion of the 60-10 ppm region of ^{13}C NMR spectrum of aspkyncin in DMSO-d_6.

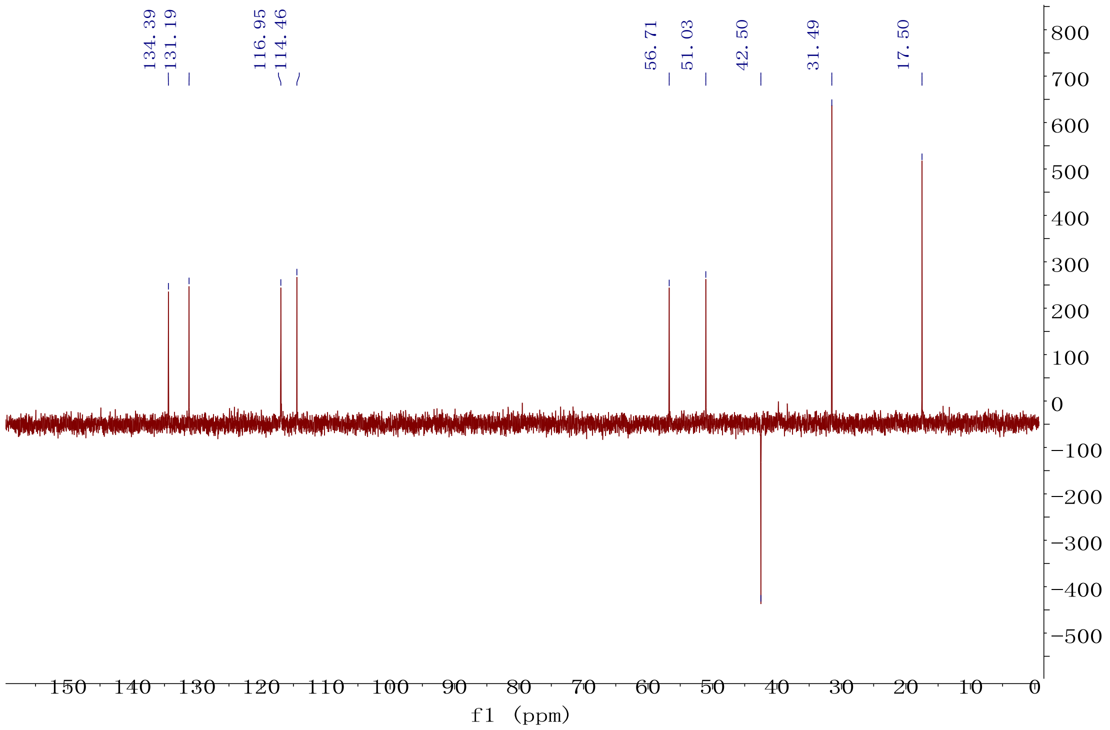

Figure S16. DEPT 135° spectrum of aspkyncin in DMSO-d_6.

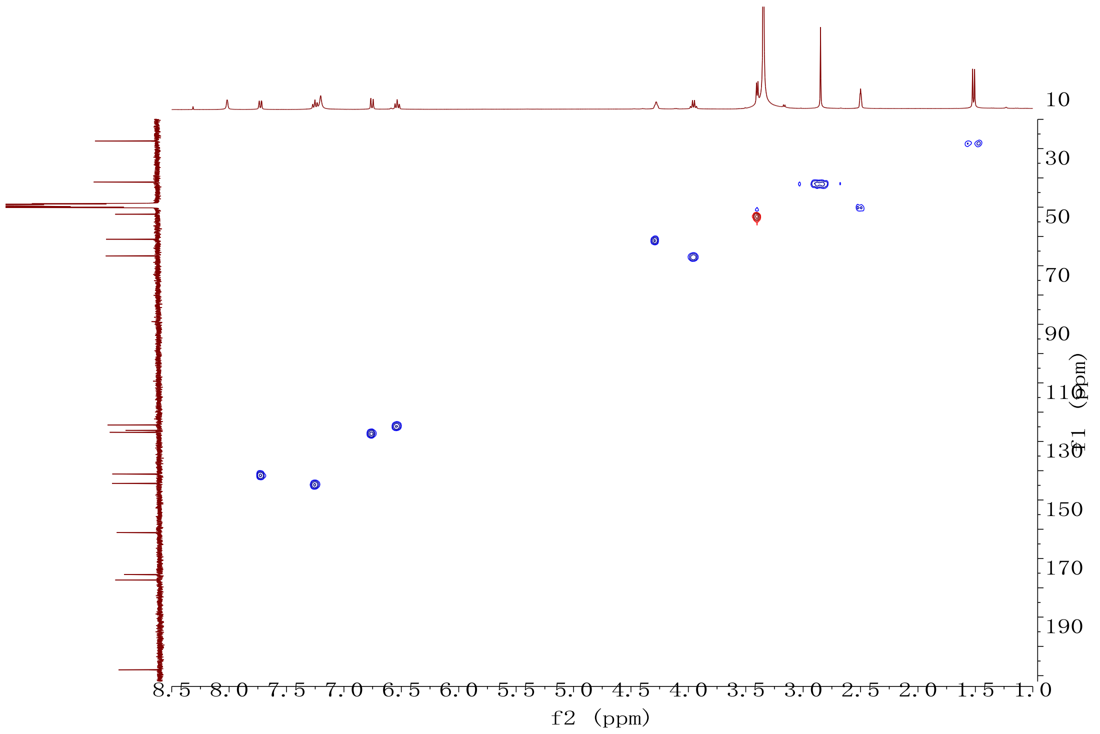

Figure S17. ^{1}H-^{13}C HSQC spectrum of aspkyncin in DMSO-d_6.

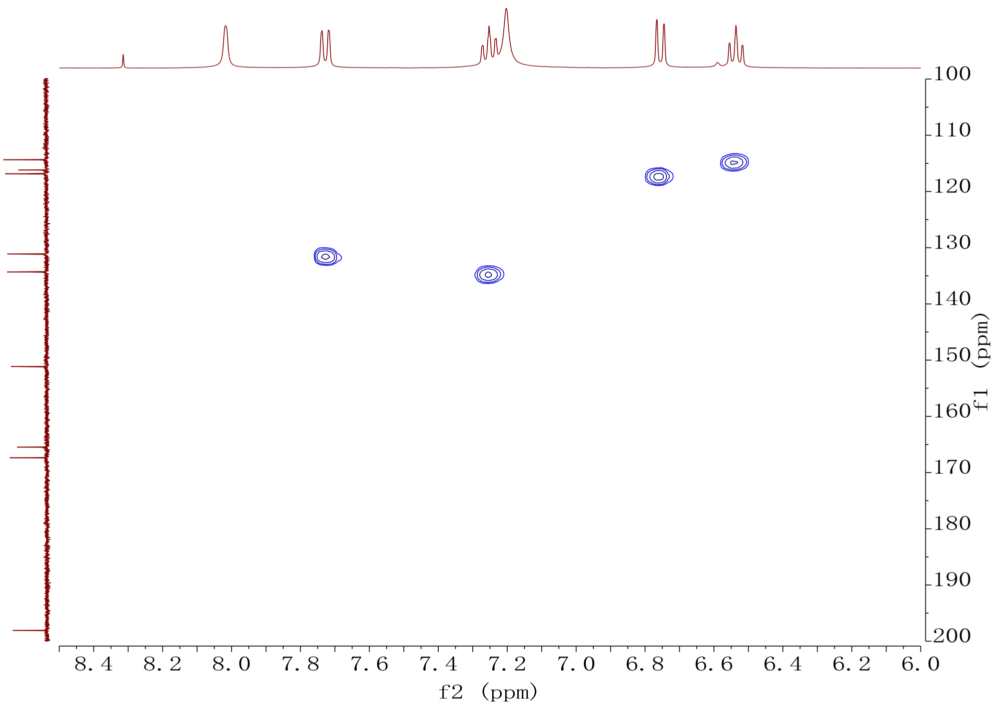

Figure S18. Expansion of the 8.5-6.0 ppm (^{1}H) and 200-100 ppm (^{13}C) region of ^{1}H-^{13}C HSQC spectrum of aspkyncin in DMSO-d_6.

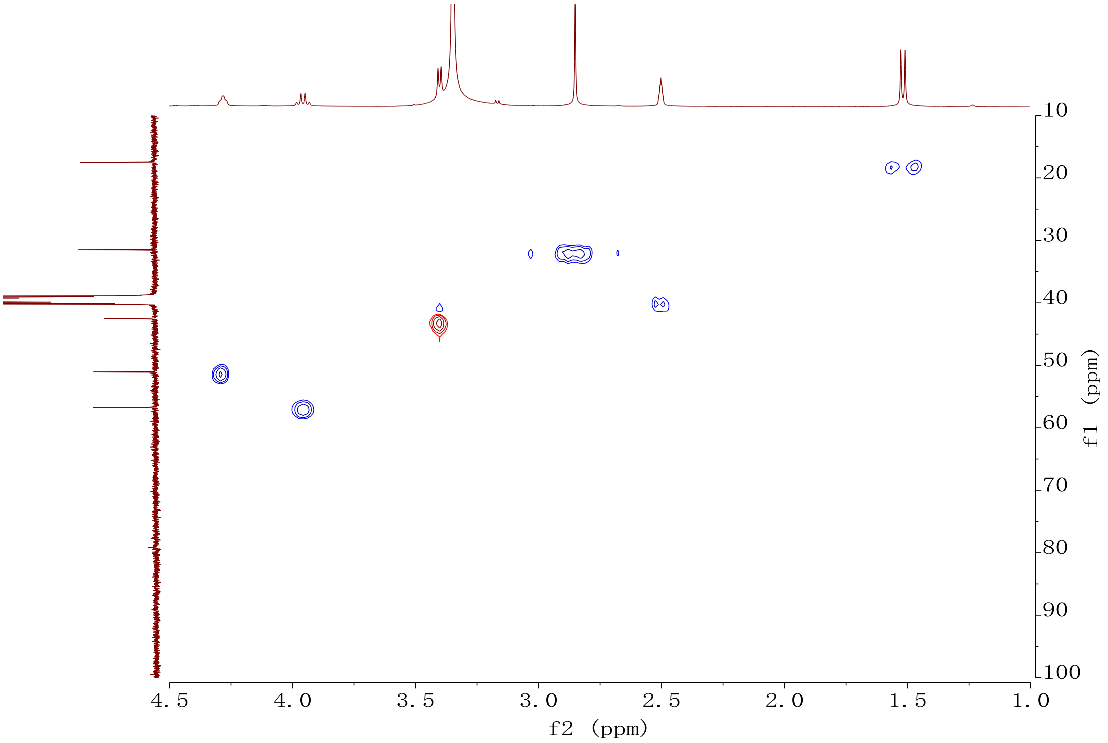

Figure S19. Expansion of the 4.5-1.0 ppm (^{1}H) and 100-10 ppm (^{13}C) region of ^{1}H-^{13}C HSQC spectrum of aspkyncin in DMSO-d_6.

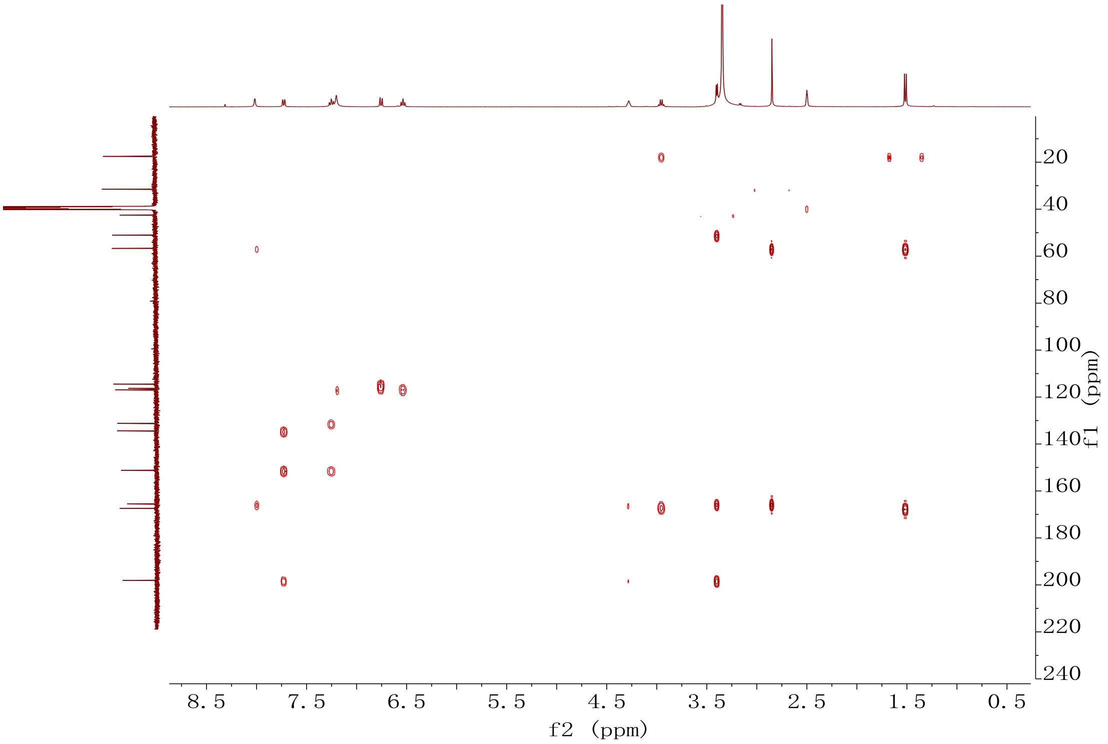

Figure S20. ^{1}H-^{13}C HMBC spectrum of aspkyncin in DMSO-d_6.

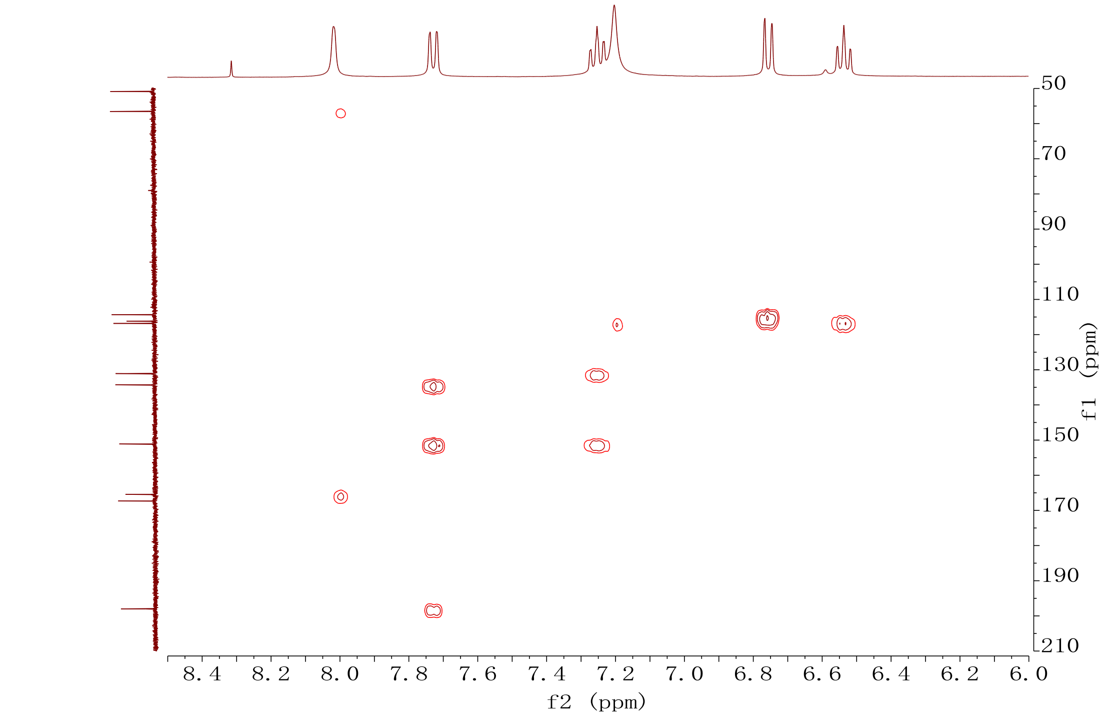

Figure S21. Expansion of the 8.5-6.0 ppm (^{1}H) and 210-50 ppm (^{13}C) region of ^{1}H-^{13}C HMBC spectrum of aspkyncin in DMSO-d_6.

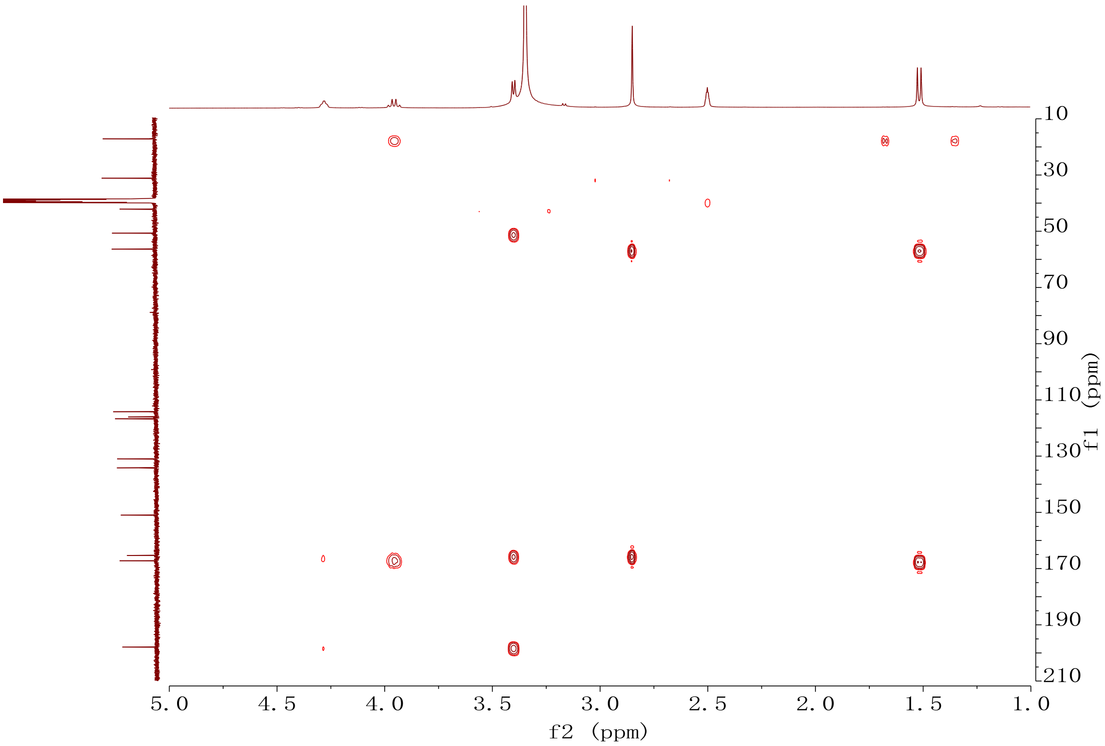

Figure S22. Expansion of the 5.0-1.0 ppm (^{1}H) and 210-10 ppm (^{13}C) region of ^{1}H-^{13}C HMBC spectrum of aspkyncin in DMSO-d_6.

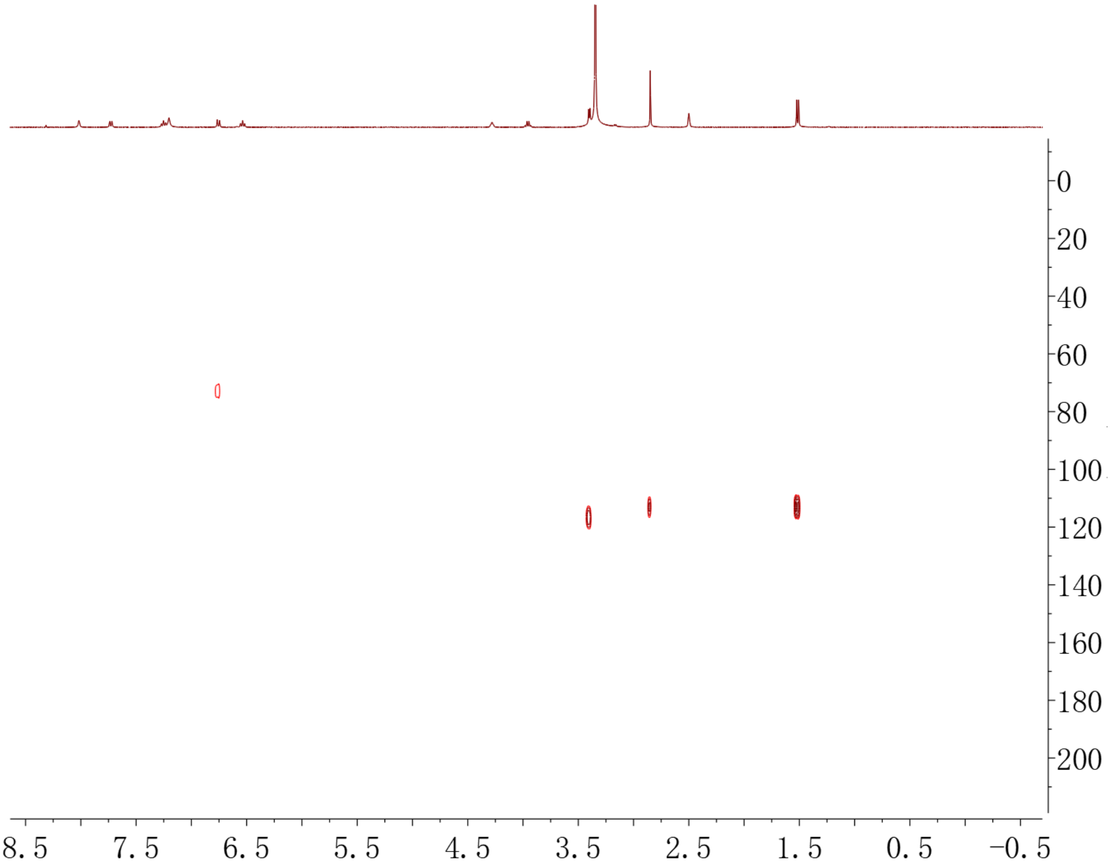

Figure S23. ^{1}H-^{15}N HMBC spectrum of aspkyncin in DMSO-d_6.

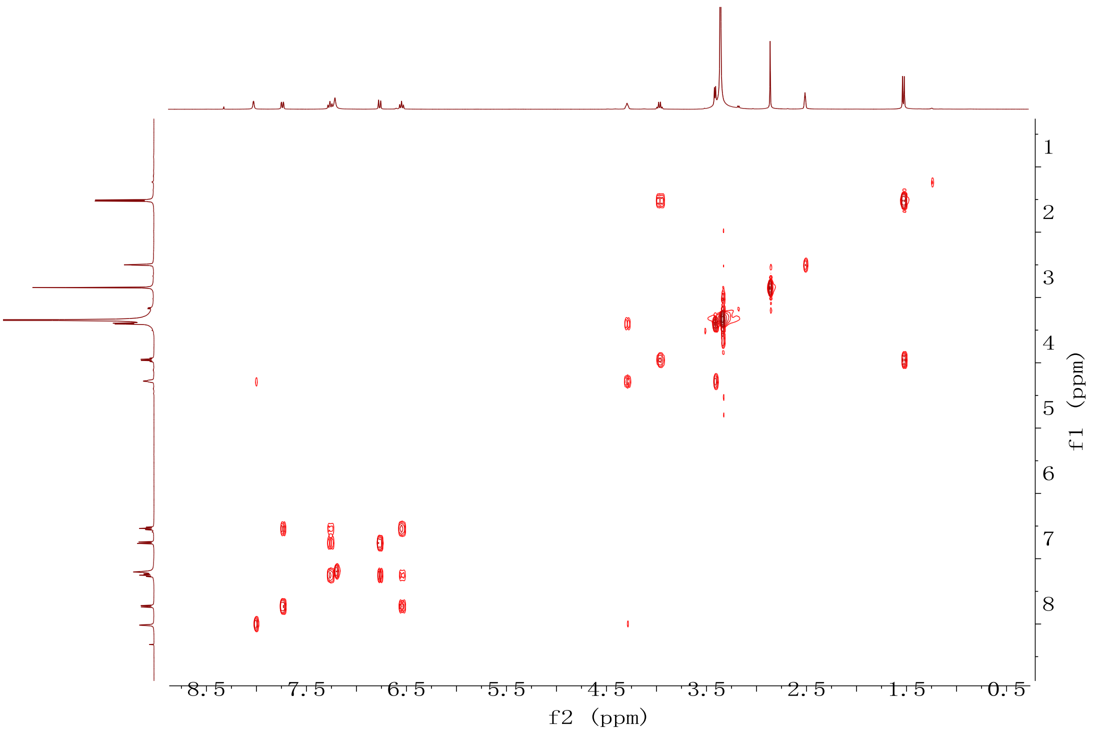

Figure S24. ^{1}H-^{1}H COSY spectrum of aspkyncin in DMSO-d_6.

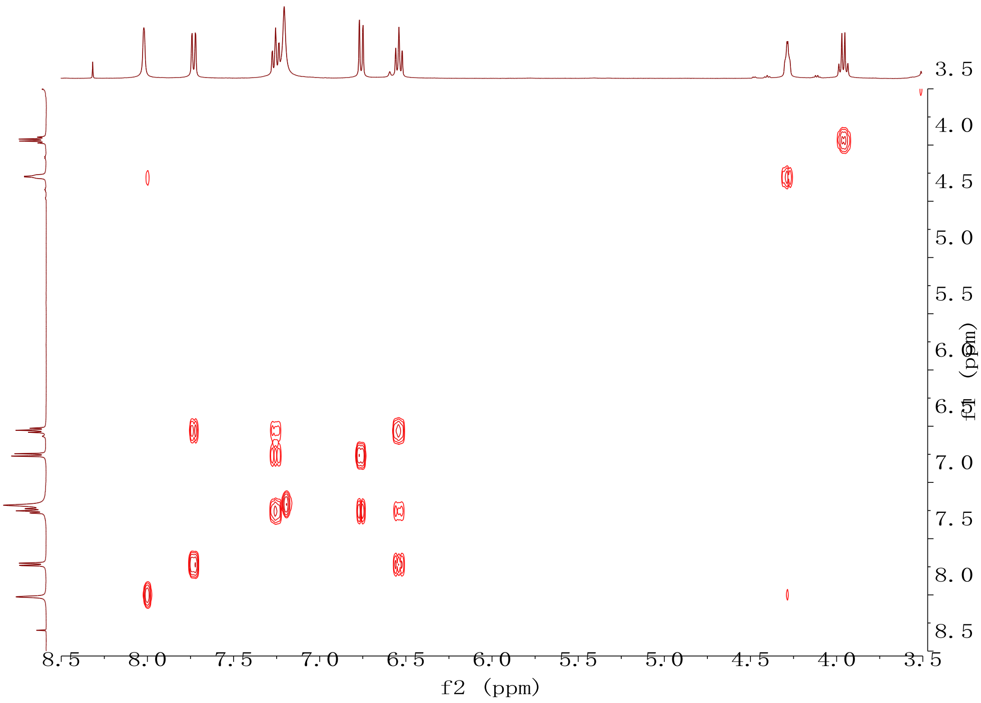

Figure S25. Expansion of the 8.5-3.5 ppm region of ^{1}H-^{1}H COSY spectrum of aspkyncin in DMSO-d_6.

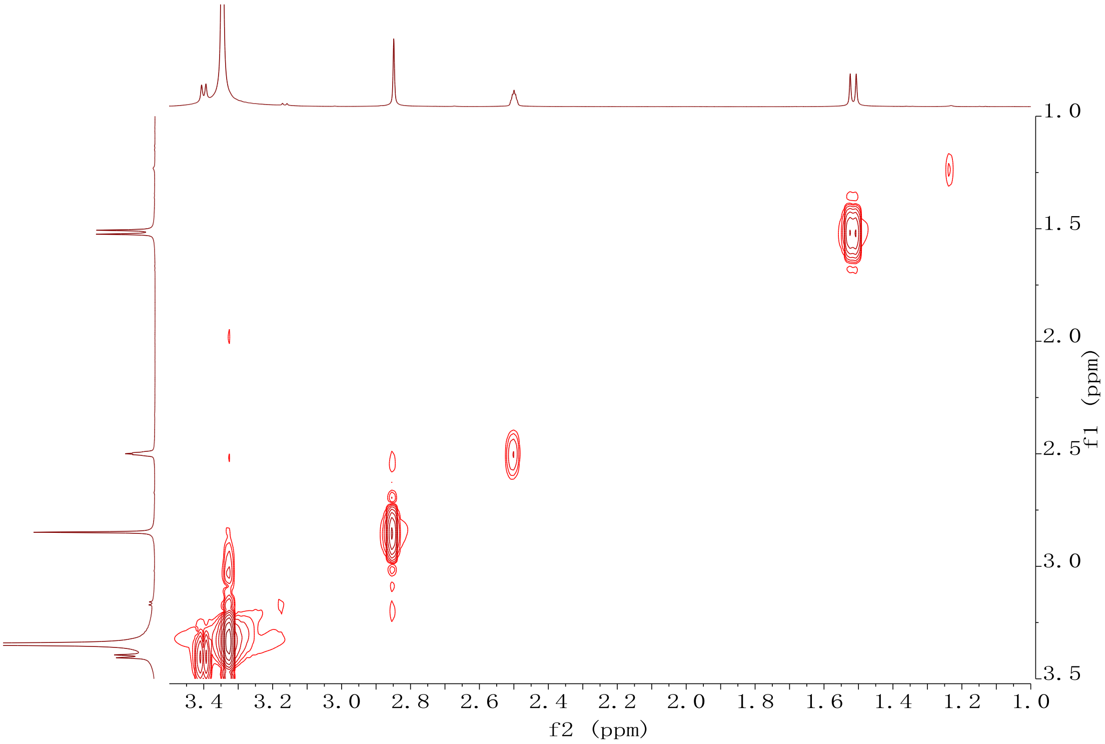

Figure S26. Expansion of the 3.5-1.0 ppm region of ^{1}H-^{1}H COSY spectrum of aspkyncin in DMSO-d_6.

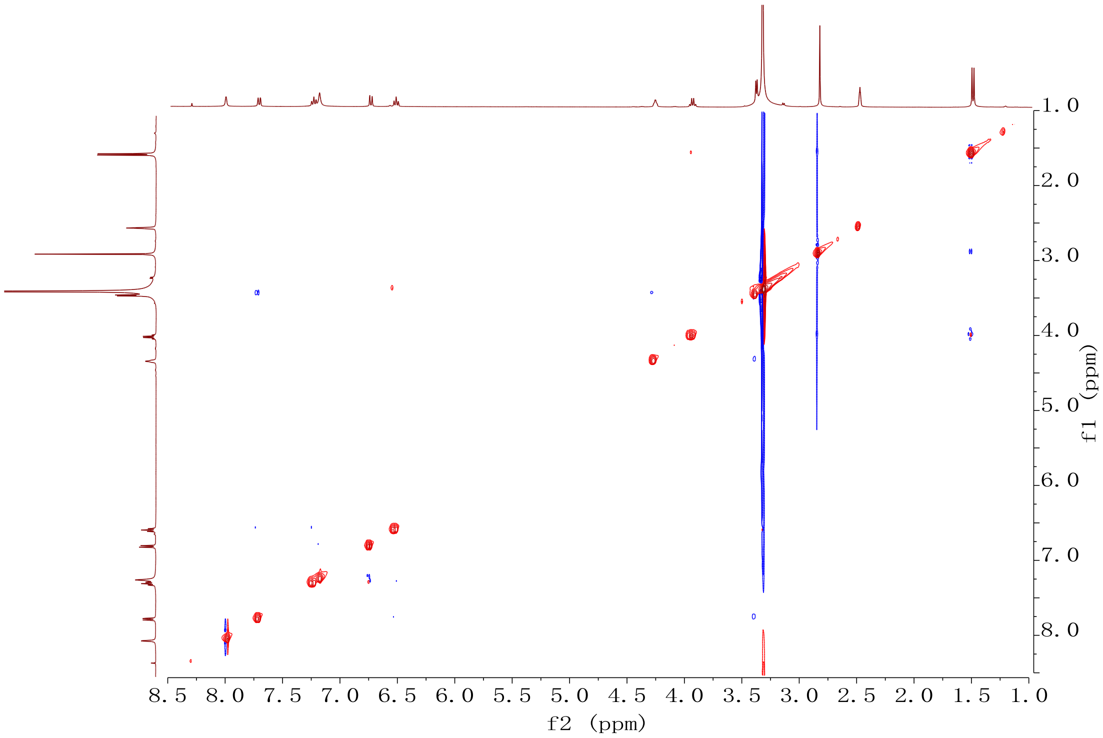

Figure S27. ^{1}H-^{1}H NOESY spectrum of aspkyncin in DMSO-d_6.

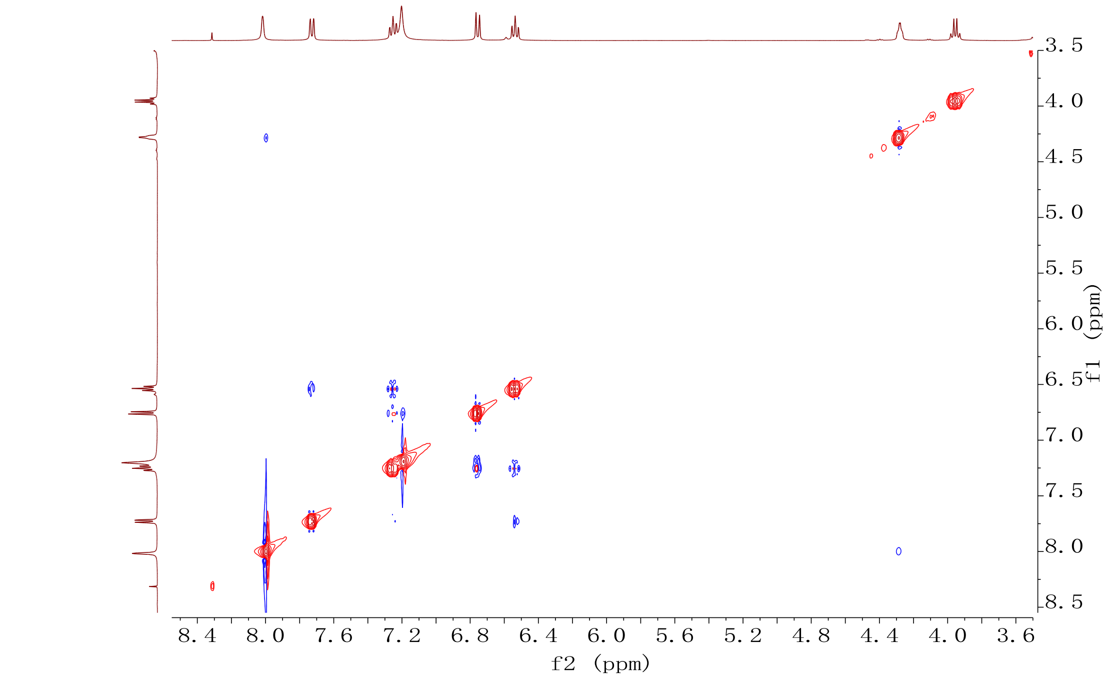

Figure S28. Expansion of the 8.5-3.5 ppm region of ^{1}H-^{1}H NOESY spectrum of aspkyncin in DMSO-d_6.

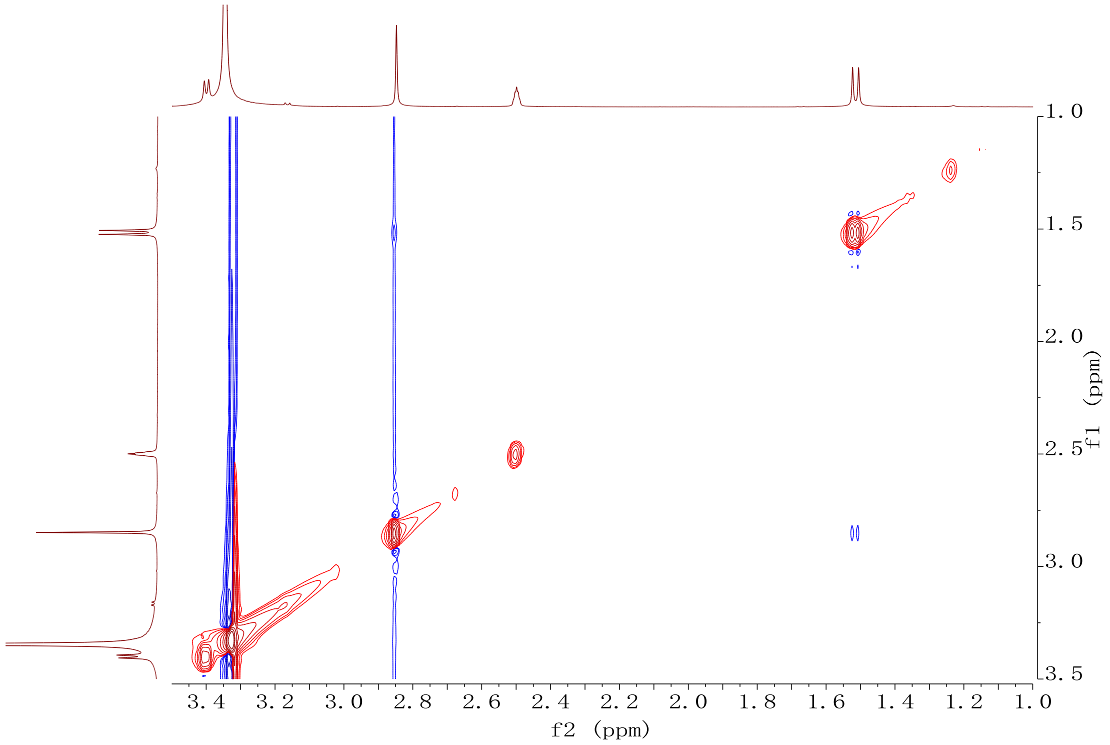

Figure S29. Expansion of the 3.5-1.0 ppm region of ^{1}H-^{1}H NOESY spectrum of aspkyncin in DMSO-d_6.
